# Supplementary figures and images for: Epigenomic regulation of human T-cell leukemia virus by chromatin-insulator CTCF
Source: PLoS Pathog. 2021 May 21;17(5):e1009577. doi: 10.1371/journal.ppat.1009577 (PMC8174705; doi:10.1371/journal.ppat.1009577)

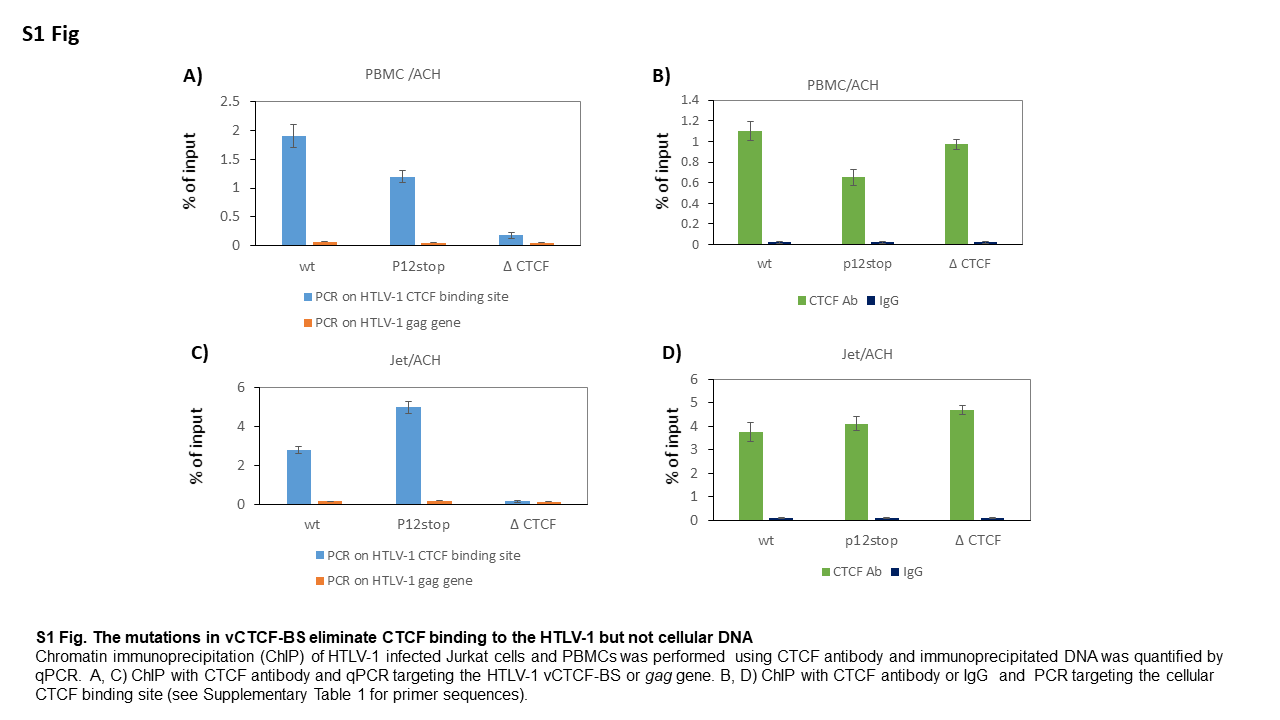

Supplement: S1 Fig — Chromatin immunoprecipitation (ChIP) of HTLV-1 infected Jurkat cells and PBMCs was performed using CTCF antibody and immunoprecipitated DNA was quantified by qPCR. A, C) ChIP with CTCF antibody and qPCR targeting the HTLV-1 vCTCF-BS or gag gene. B, D) ChIP with CTCF antibody or IgG and PCR targeting the cellular CTCF binding site (see S3 Table for primer sequences). (TIF) [file ppat.1009577.s001.TIF]

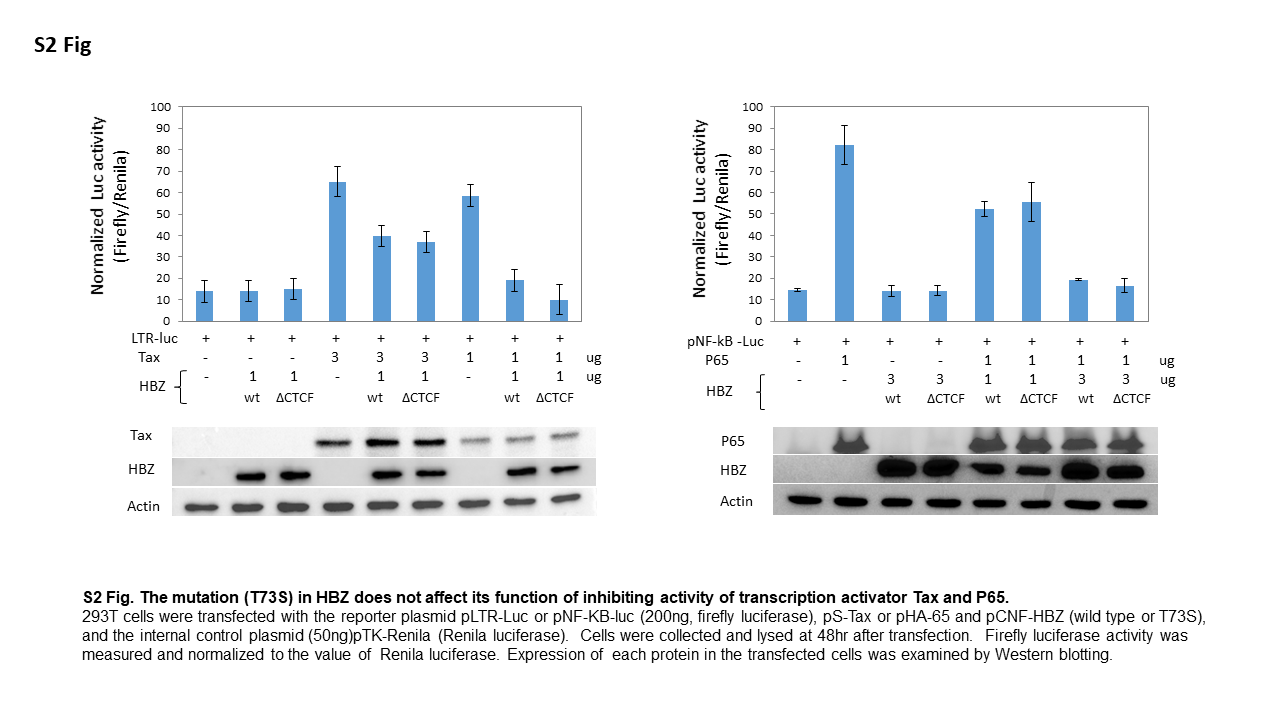

Supplement: S2 Fig — 293T cells were transfected with the reporter plasmid pLTR-Luc or pNF-KB-luc (200 ng, firefly luciferase), pS-Tax or pHA-65 and pCNF-HBZ (wild type or T73S), and the internal control plasmid (50 ng) pTK-Renila (Renila luciferase). Cells were collected and lysed at 48 hr after transfection. Firefly luciferase activity was measured and normalized to the value of Renila luciferase. Expression of each protein in the transfected cells was examined by Western blotting. (TIF) [file ppat.1009577.s002.TIF]

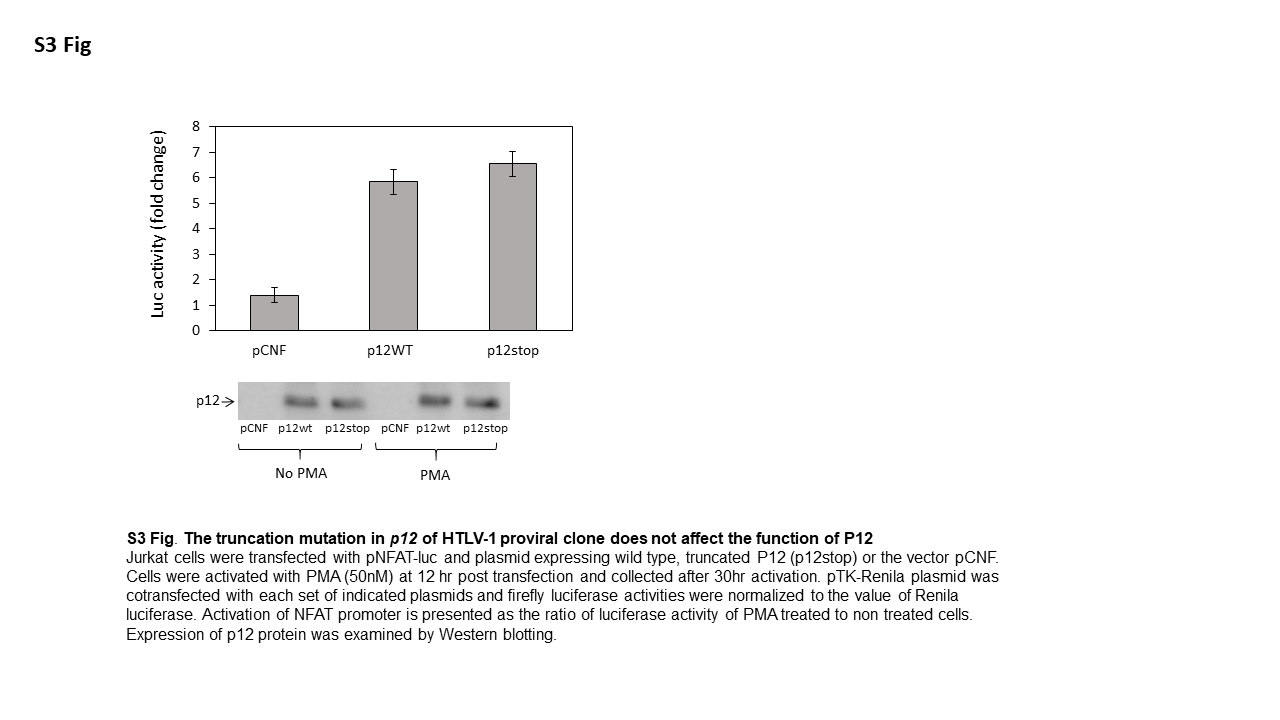

Supplement: S3 Fig — Jurkat cells were transfected with pNFAT-luc and plasmid expressing wild type, truncated P12 (p12stop) or the vector pCNF. Cells were activated with PMA (50 nM) at 12 hr post transfection and collected after 30 hr activation. pTK-Renila plasmid was cotransfected with each set of indicated plasmids and firefly luciferase activities were normalized to the value of Renila luciferase. Activation of NFAT promoter is presented as the ratio of luciferase activity of PMA treated to non treated cells. Expression of p12 protein was examined by Western blotting. (TIF) [file ppat.1009577.s003.TIF]

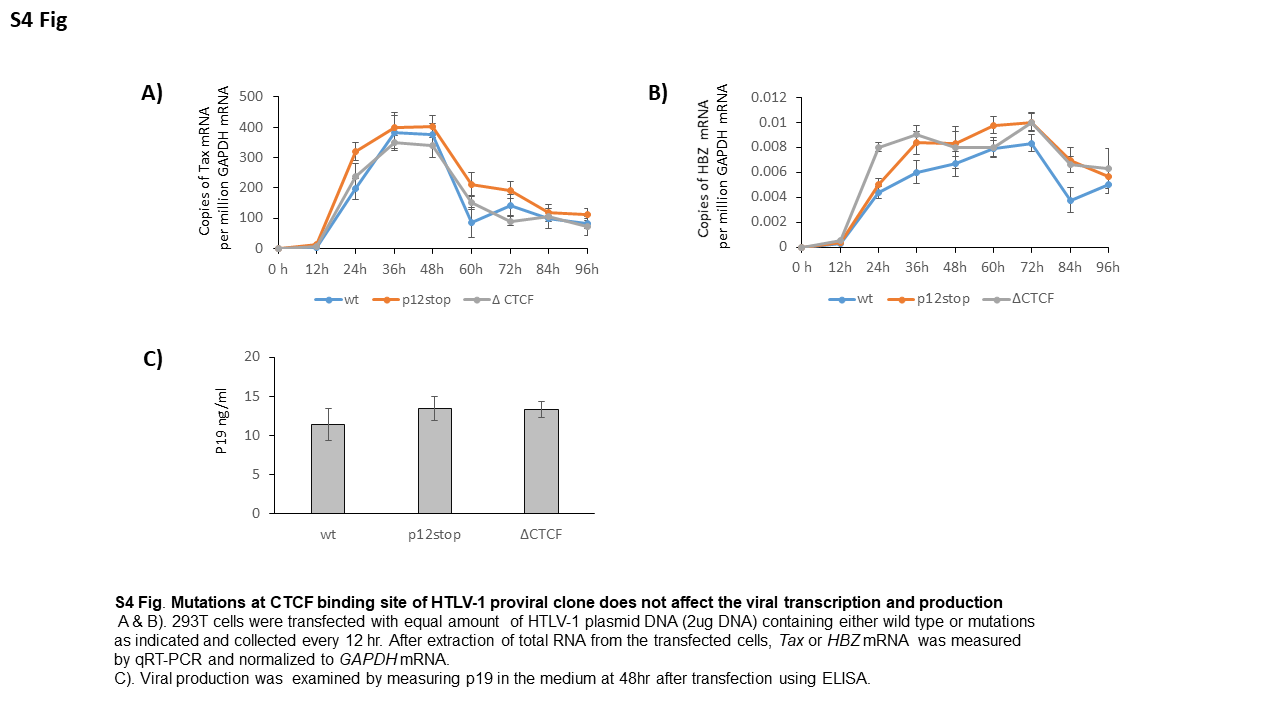

Supplement: S4 Fig — A & B). 293T cells were transfected with equal amounts of HTLV-1 plasmid DNA (2 ug DNA) containing either wild type or mutations as indicated and collected every 12 hr. After extraction of total RNA from the transfected cells, Tax or hbz mRNA was measured by qRT-PCR and normalized to GAPDH mRNA. C). Viral production was examined by measuring p19 in the medium at 48hr after transfection using ELISA. (TIF) [file ppat.1009577.s004.TIF]

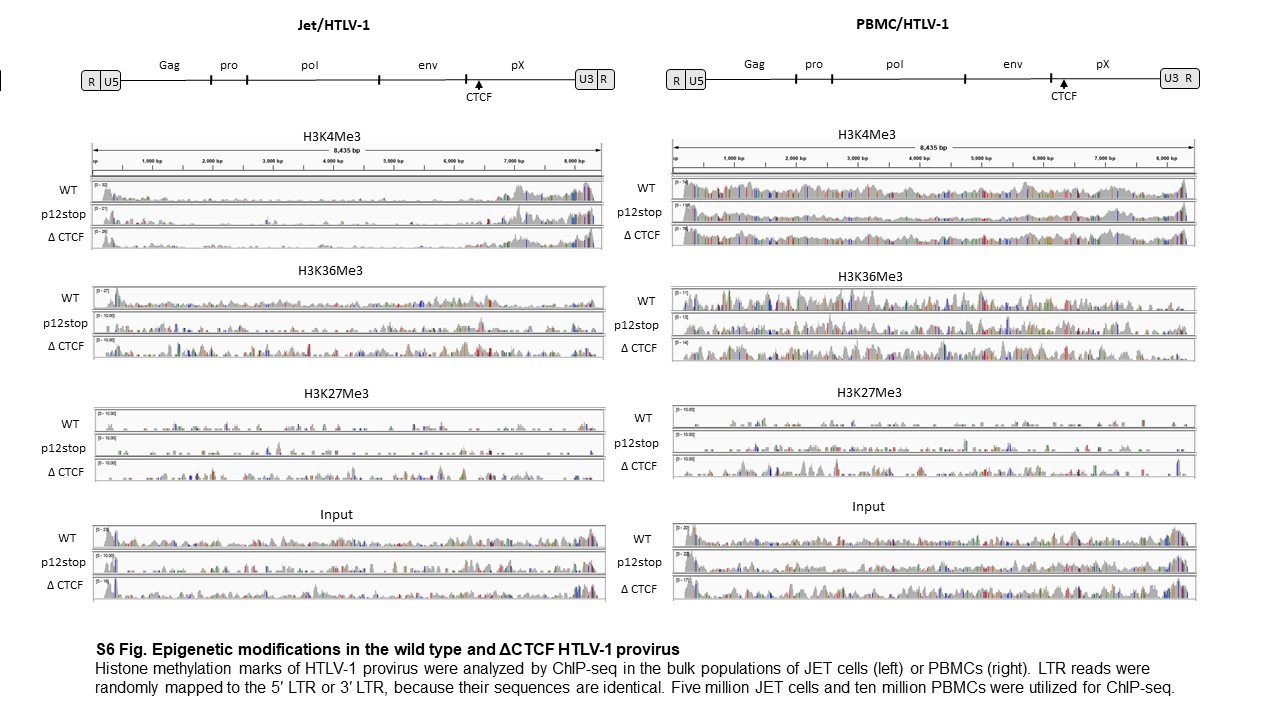

Supplement: S6 Fig — Histone methylation marks of HTLV-1 provirus were analyzed by ChIP-seq in the bulk populations of JET cells (left) or PBMCs (right). LTR reads were randomly mapped to the 5′ LTR or 3′ LTR, because their sequences are identical. Five million JET cells and ten million PBMCs were utilized for ChIP-seq. (TIF) [file ppat.1009577.s006.TIF]

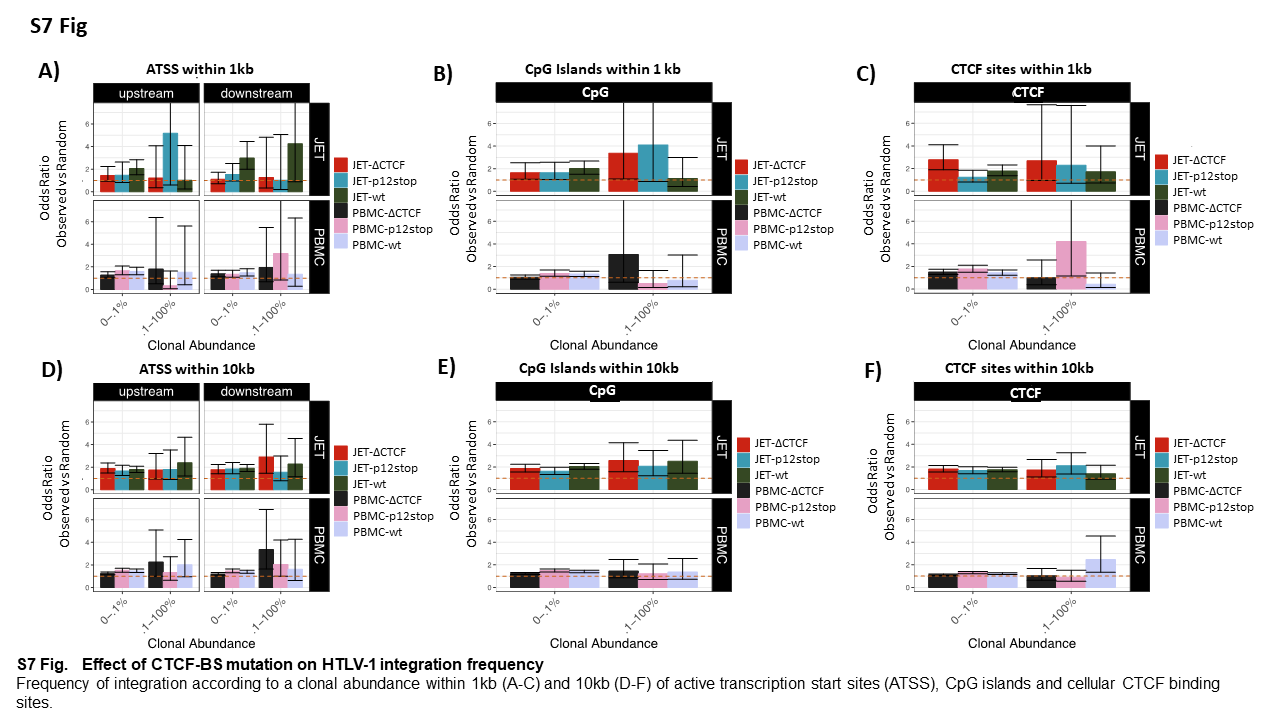

Supplement: S7 Fig — Frequency of integration according to a clonal abundance within 1 kb (A-C) and 10 kb (D-F) of active transcription start sites (ATSS), CpG islands and cellular CTCF binding sites. (TIF) [file ppat.1009577.s007.TIF]

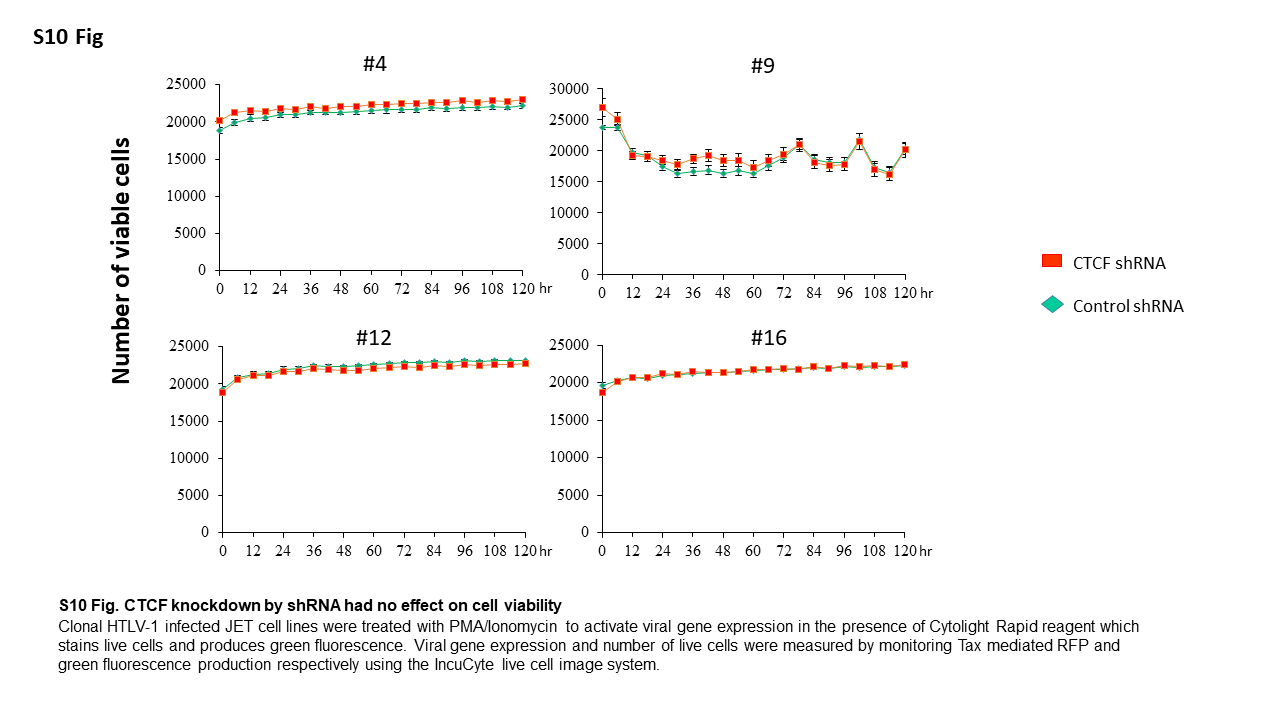

Supplement: S10 Fig — Clonal HTLV-1 infected JET cell lines were treated with PMA/Ionomycin to activate viral gene expression in the presence of Cytolight Rapid reagent which stains live cells and produces green fluorescence. Viral gene expression and number of live cells were measured by monitoring Tax mediated RFP and green fluorescence product, respectively using the IncuCyte live cell image system. (TIF) [file ppat.1009577.s010.TIF]

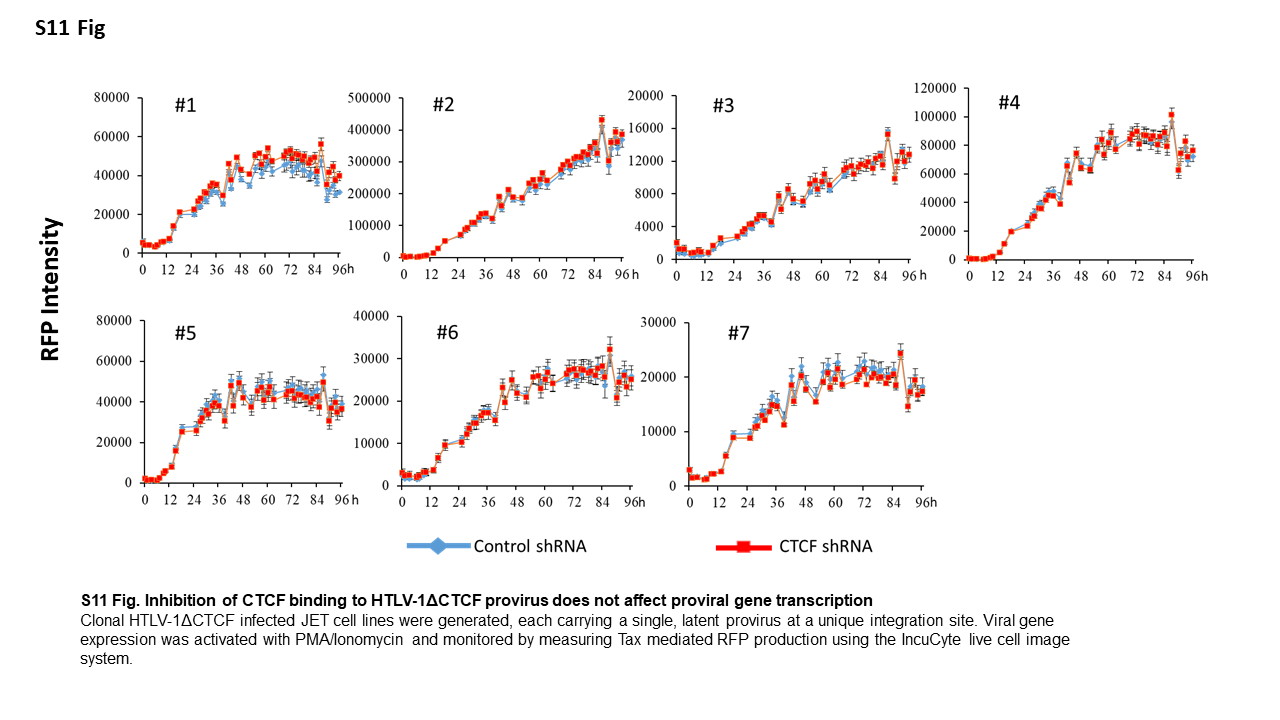

Supplement: S11 Fig — Clonal HTLV-1ΔCTCF infected JET cell lines were generated, each carrying a single, latent provirus at a unique integration site. Viral gene expression was activated with PMA/Ionomycin and monitored by measuring Tax mediated RFP production using the IncuCyte live cell image system. (TIF) [file ppat.1009577.s011.TIF]

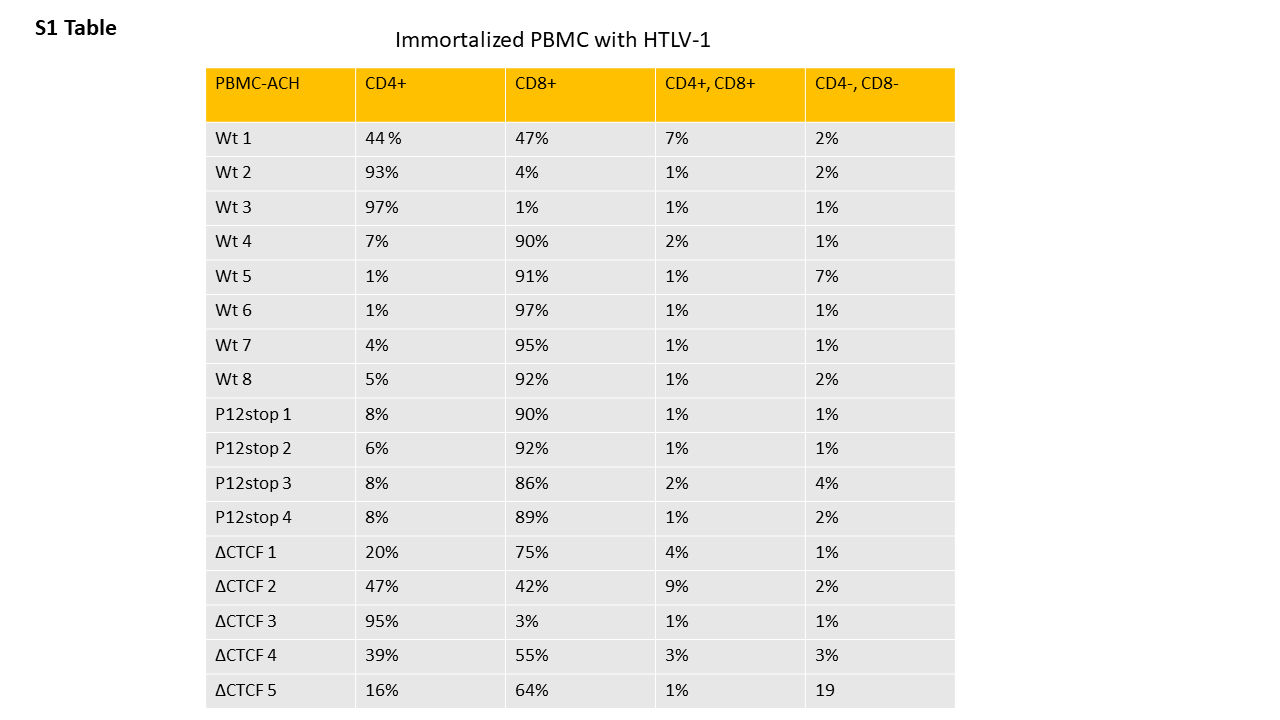

Supplement: S1 Table — (TIF) [file ppat.1009577.s013.TIF]

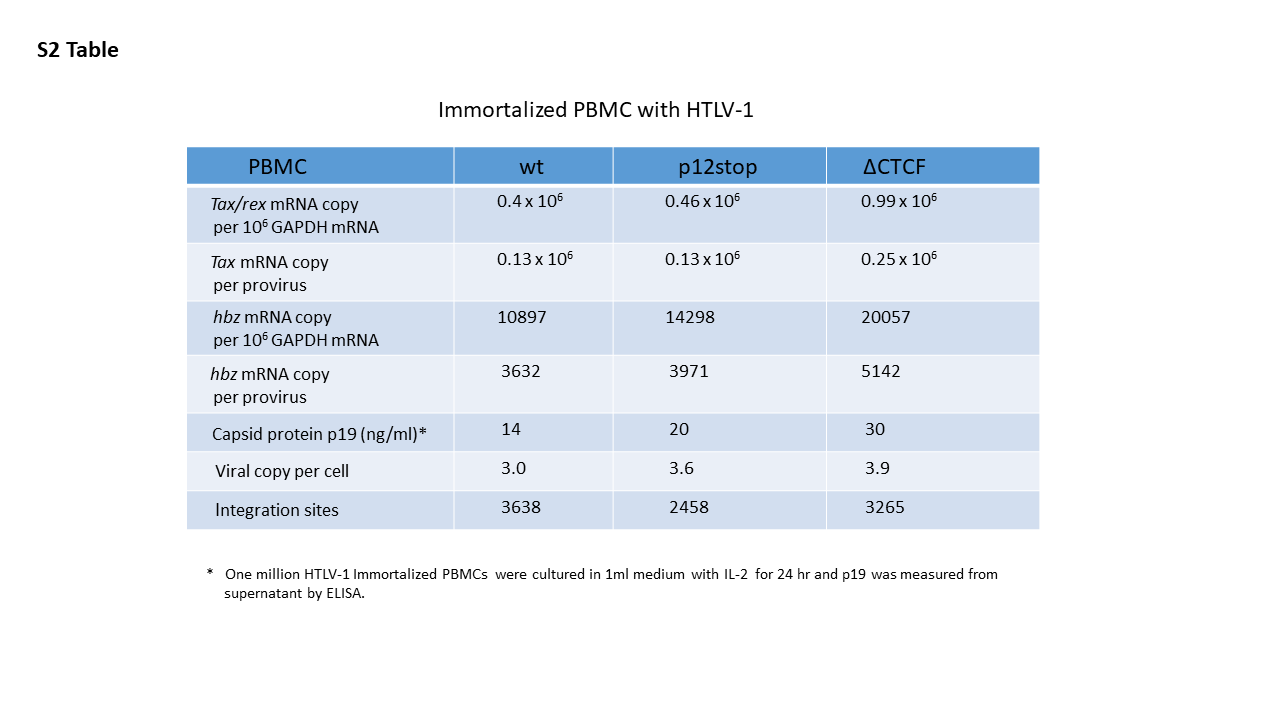

Supplement: S2 Table — (TIF) [file ppat.1009577.s014.TIF]

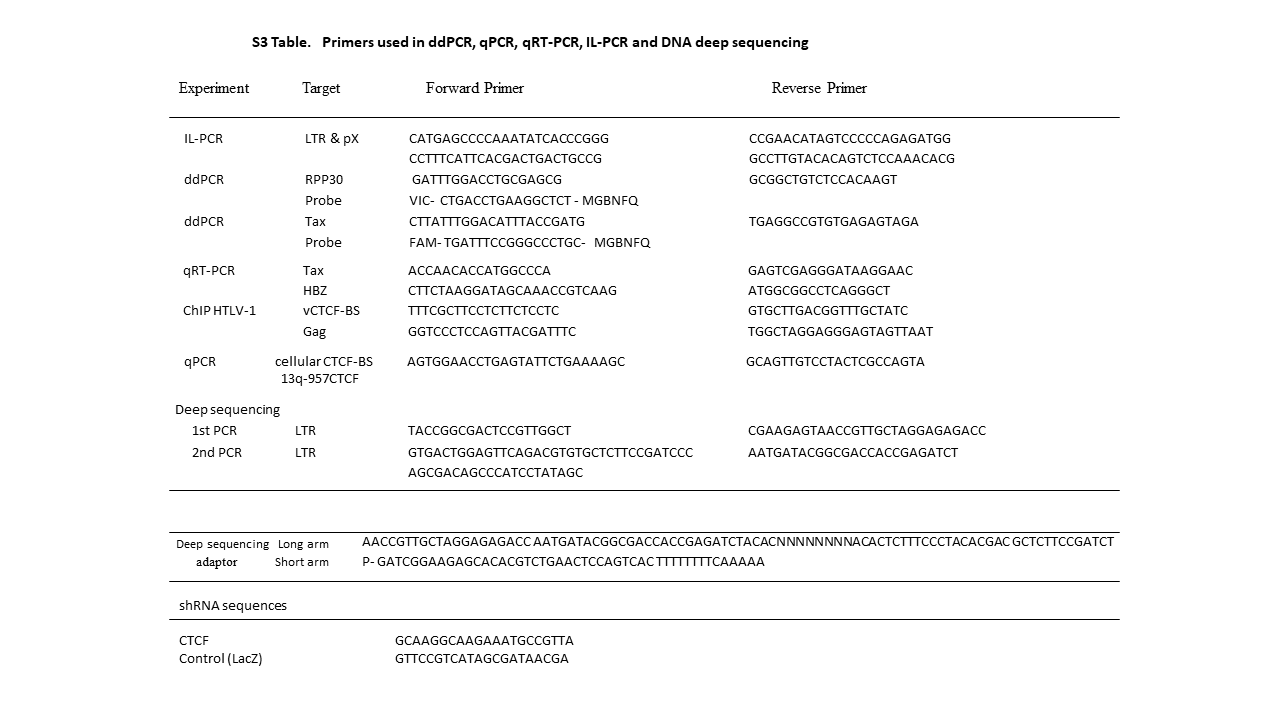

Supplement: S3 Table — (TIF) [file ppat.1009577.s015.TIF]

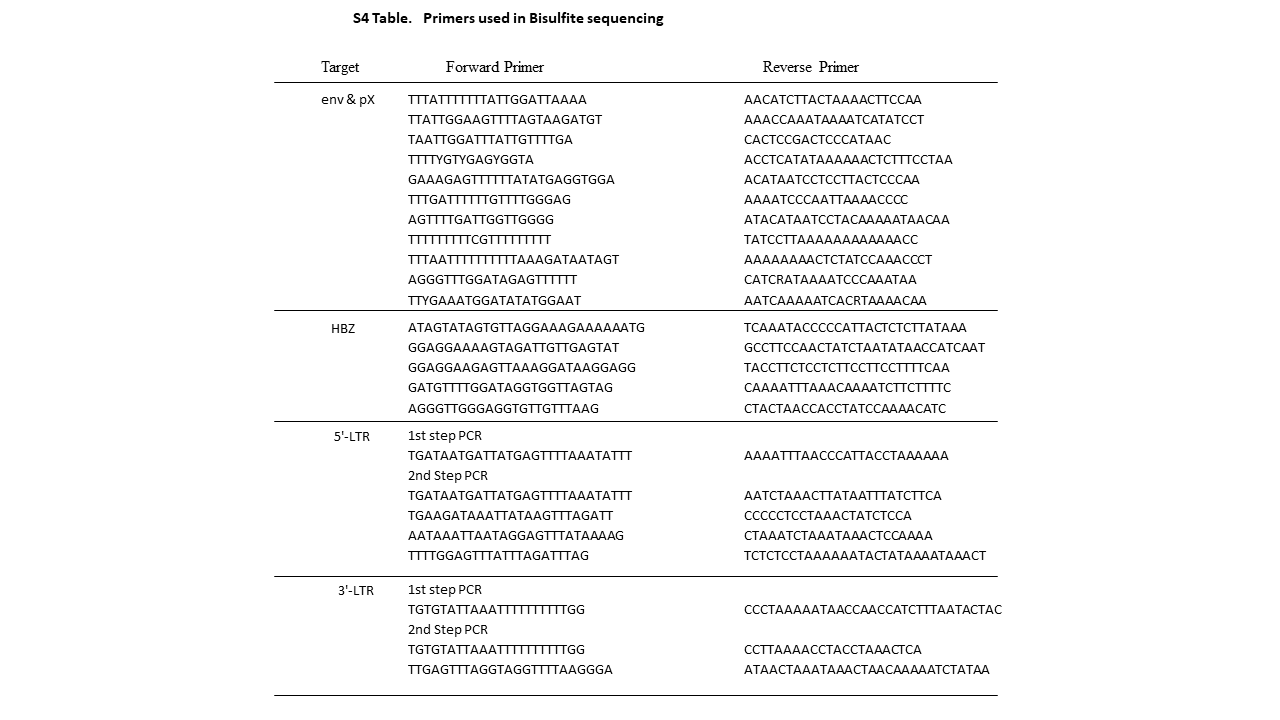

Supplement: S4 Table — (TIF) [file ppat.1009577.s016.TIF]
