# Supplementary material for: Epigenomic regulation of human T-cell leukemia virus by chromatin-insulator CTCF
Source: PLoS Pathog. 2021 May 21;17(5):e1009577. doi: 10.1371/journal.ppat.1009577 (PMC8174705; doi:10.1371/journal.ppat.1009577)
Supplement: S5 Fig — A, D). DNA methylation is presented as the percentage of methylated CpG (Y- axis) in 5’LTR (A), 3’LTR and hbz on anti-sense strand (D) of HTLV-1ΔCTCF compared to wild type HTLV-1 or HTLV-1p12stop provirus at the indicated locations of the viral DNA (X- axis). Upper panel: HTLV-1 immortalized PBMCs; lower panel: HTLV-1 infected JET cells. B, C). DNA methylation is shown as a fold increase of methylated CpG (Y- axis) in 5’LTR of HTLV-1ΔCTCF HTLV-1 compared to wild type HTLV-1 or HTLV-1p12stop provirus in PBMCs (B) and JET cells (C). E, F). DNA methylation is shown as a fold increase of methylated CpG in 3’LTR and hbz on anti-sense strand of HTLV-1ΔCTCF compared to wild type HTLV-1 or HTLV-1p12stop provirus in PBMCs (E) and JET cells (F). Upper panel: 3’LTR; lower panel: hbz. CTCF binding site: 7041-7052 as indicated by an arrow. * Lost CpG sites, ** New CpG site due to the introduced mutations in vCTCF-BS. (PDF) [file ppat.1009577.s005.pdf]

S5A Fig. Methylation in 5' LTR region (Sense strand)

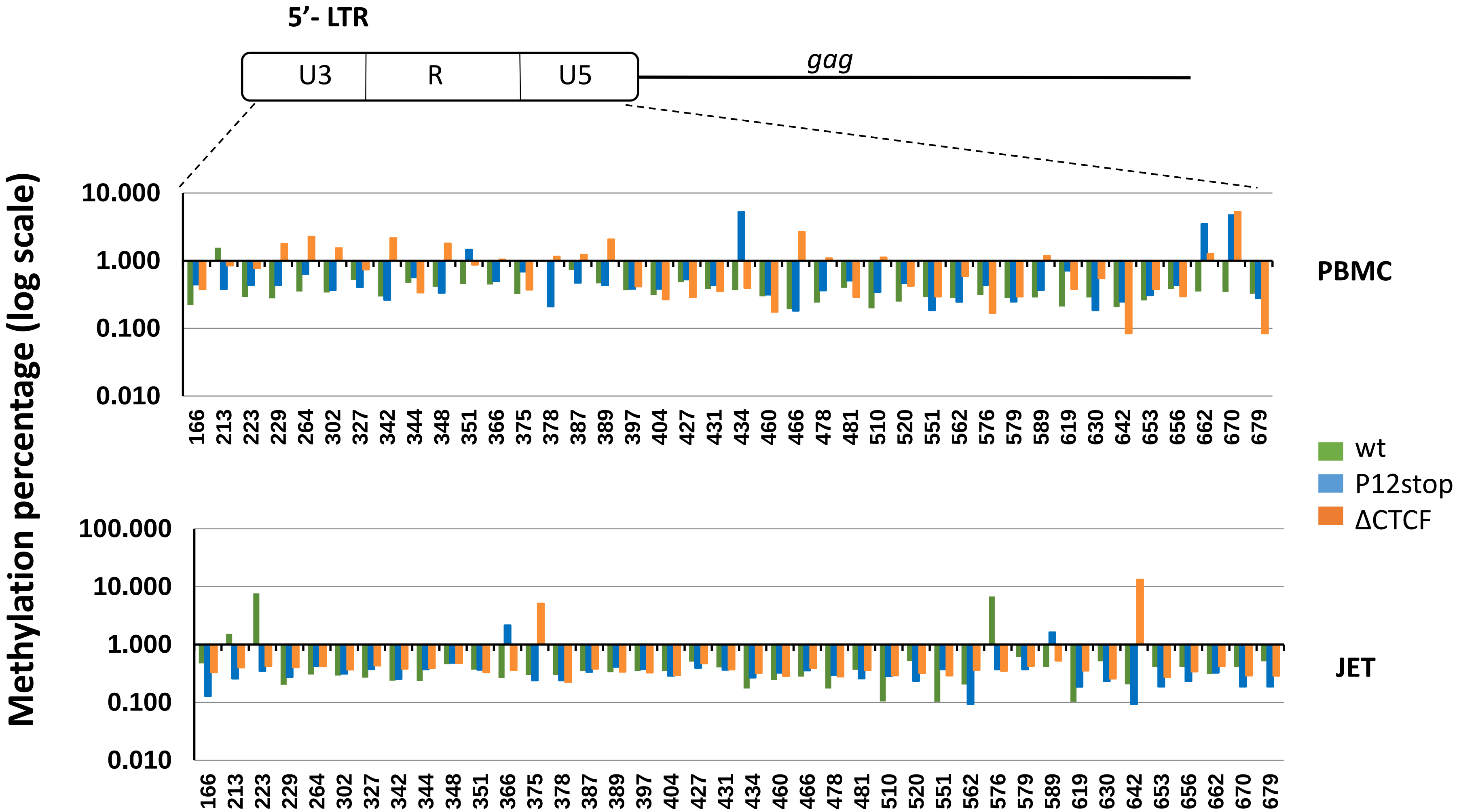

S5B and 5C Fig

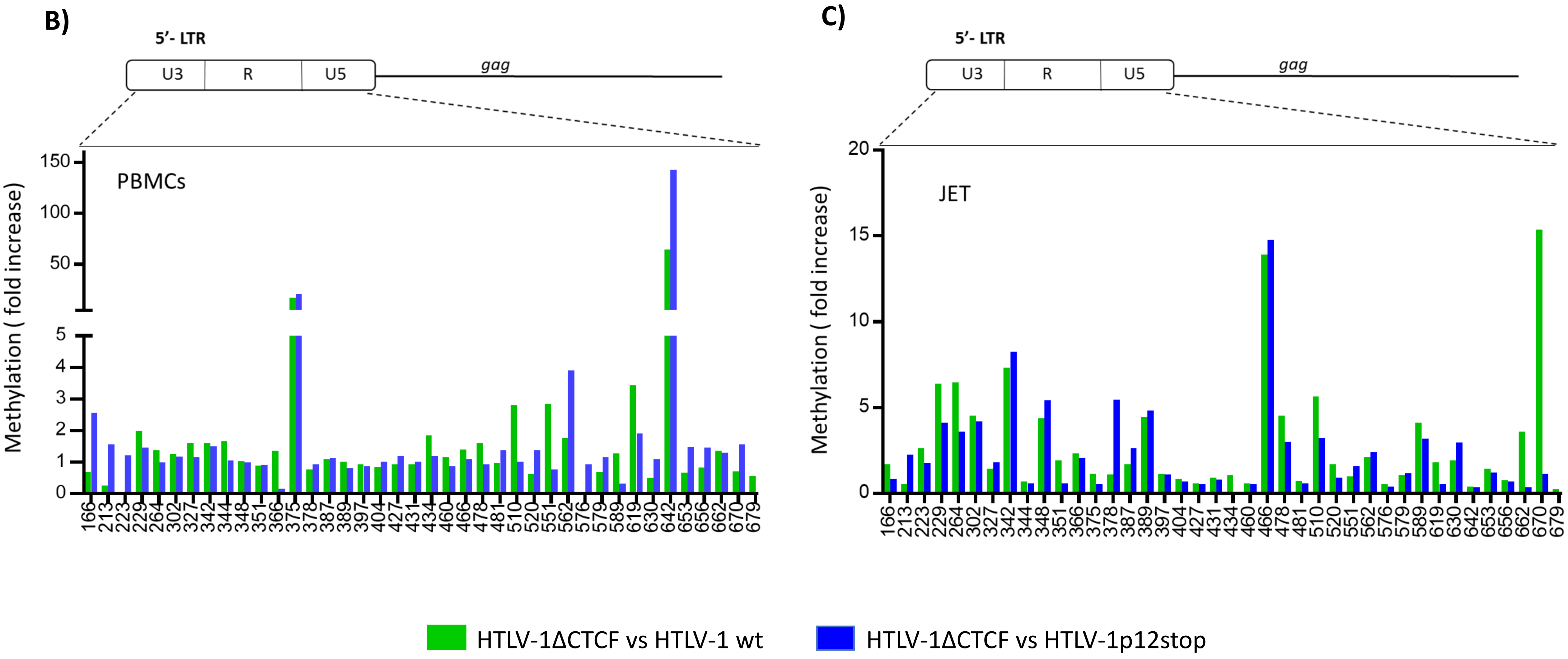

S5D Fig. Methylation in 3'-LTR region (antisense strand)

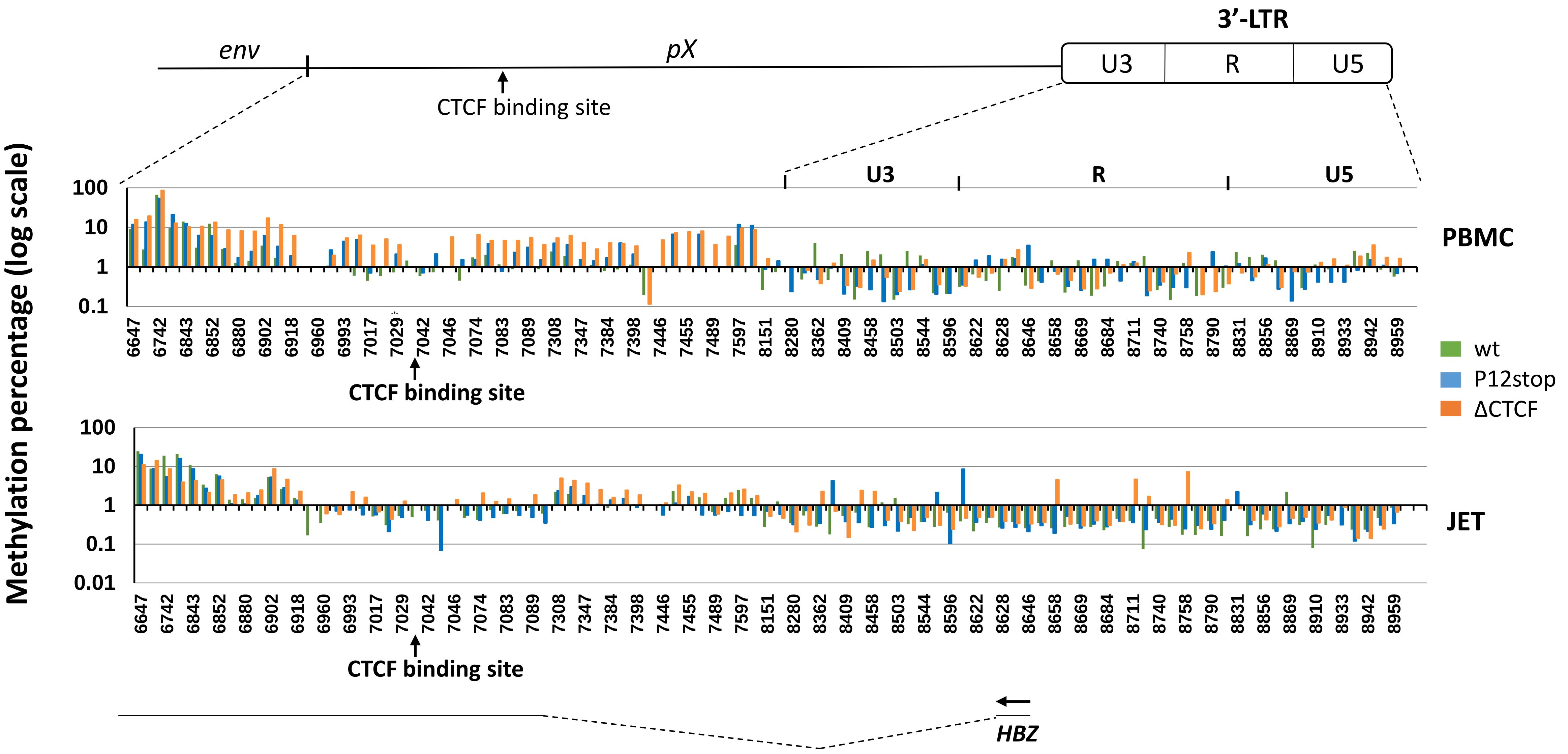

S5E Fig

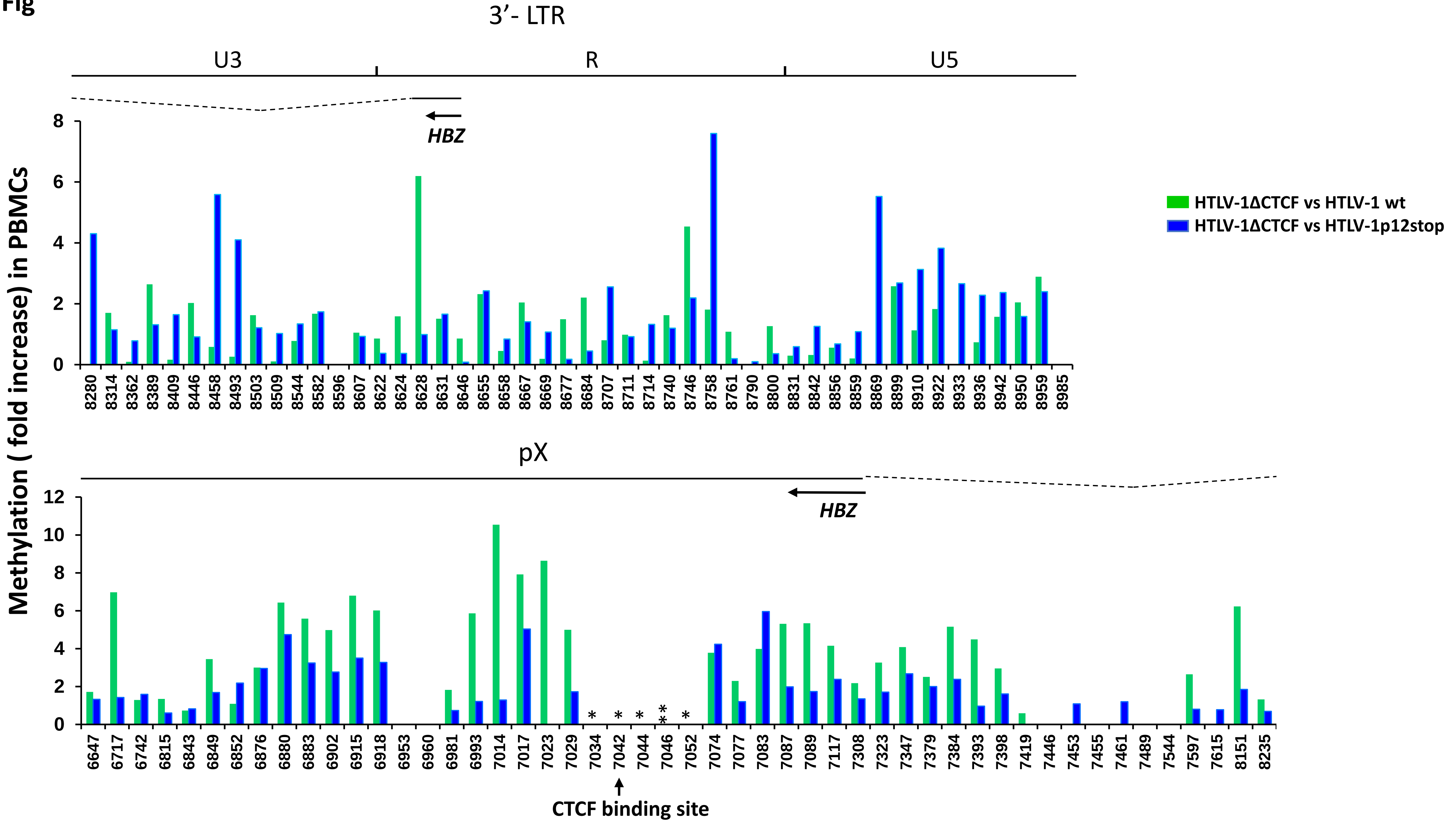

3'- LTR
U3
R
U5
HBZ
Methylation (fold increase) in PBMCs
HTLV-1ΔCTCF vs HTLV-1 wt
HTLV-1ΔCTCF vs HTLV-1p12stop
8280
8314
8362
8389
8409
8446
8458
8493
8503
8509
8544
8582
8596
8607
8622
8624
8628
8631
8646
8655
8658
8667
8669
8677
8684
8707
8711
8714
8740
8746
8758
8761
8790
8800
8831
8842
8856
8859
8869
8899
8910
8922
8933
8936
8942
8950
8959
8985
pX
HBZ
6647
6717
6742
6815
6843
6849
6852
6876
6880
6883
6902
6915
6918
6953
6960
6981
6993
7014
7017
7023
7029
7034
7042
7044
7046
7052
7074
7077
7083
7087
7089
7117
7308
7323
7347
7379
7384
7393
7398
7419
7446
7453
7455
7461
7489
7544
7597
7615
8151
8235
CTCF binding site

S5F Fig

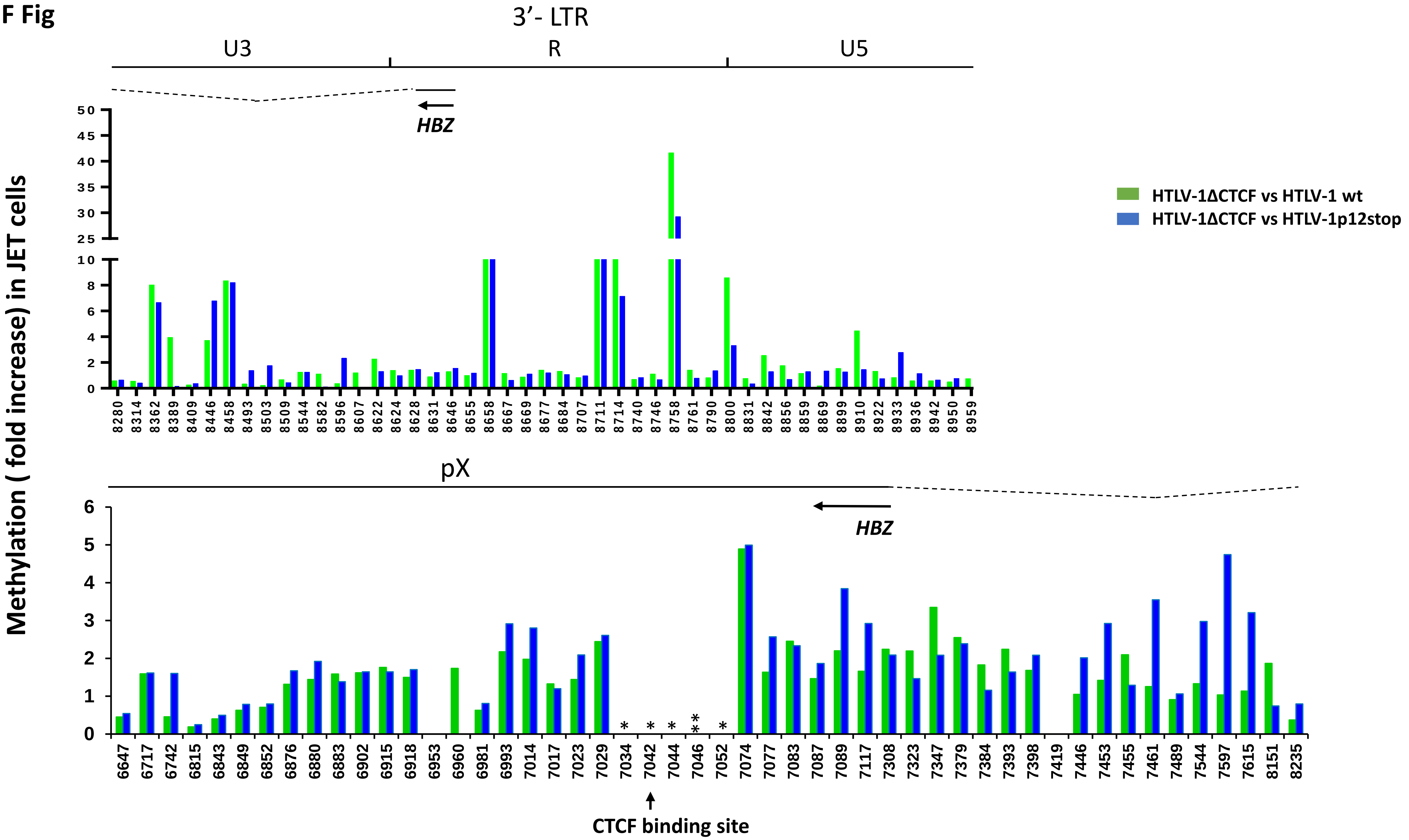
3'- LTR
U3
R
U5
HBZ
HTLV-1ΔCTCF vs HTLV-1 wt
HTLV-1ΔCTCF vs HTLV-1p12stop
Methylation (fold increase) in JET cells
8280 8314 8362 8389 8409 8446 8458 8493 8503 8509 8544 8582 8596 8607 8622 8624 8628 8631 8646 8655 8658 8667 8669 8677 8684 8707 8711 8714 8740 8746 8758 8761 8790 8800 8831 8842 8856 8859 8869 8899 8910 8922 8933 8936 8942 8950 8959
pX
HBZ
6647 6717 6742 6815 6843 6849 6852 6876 6880 6883 6902 6915 6918 6953 6960 6981 6993 7014 7017 7023 7029 7034 7042 7044 7046 7052 7074 7077 7083 7087 7089 7117 7308 7323 7347 7379 7384 7393 7398 7419 7446 7453 7455 7461 7489 7544 7597 7615 8151 8235
CTCF binding site

S5 Fig. DNA methylation change in 5’LTR and 3’LTR of the HTLV-1∆CTCF compared to wild type HTLV-1 and HTLV-1p12stop provirus

A, D). DNA methylation is presented as the percentage of methylated CpG (Y- axis) in 5’LTR (A), 3’LTR and *HBZ* on anti-sense strand (D) of HTLV-1ΔCTCF compared to wild type HTLV-1 or HTLV-1p12stop provirus at the indicated locations of the viral DNA (X- axis). Upper panel: HTLV-1 immortalized PBMCs; lower panel: HTLV-1 infected JET cells.
B, C). DNA methylation is shown as a fold increase of methylated CpG (Y- axis) in 5’LTR of HTLV-1ΔCTCF HTLV-1 compared to wild type HTLV-1 or HTLV-1p12stop provirus in PBMCs (B) and JET cells (C).
E, F). DNA methylation is shown as a fold increase of methylated CpG in 3’LTR and *HBZ* on anti-sense strand of HTLV-1ΔCTCF compared to wild type HTLV-1 or HTLV-1p12stop provirus in PBMCs (E) and JET cells (F). Upper panel: 3’LTR; lower panel: *HBZ.* CTCF binding site: 7041-7052 as indicated by an arrow. * Lost CpG sites, ** New CpG site due to the introduced mutations in vCTCF-BS.
