# Supplementary material for: Epigenomic regulation of human T-cell leukemia virus by chromatin-insulator CTCF
Source: PLoS Pathog. 2021 May 21;17(5):e1009577. doi: 10.1371/journal.ppat.1009577 (PMC8174705; doi:10.1371/journal.ppat.1009577)
Supplement: S8 Fig — Clonal HTLV-1 infected JET cell lines were generated, each carrying a single, latent provirus at a unique integration site. Viral gene expression was activated with PMA/Ionomycin and monitored by measuring Tax mediated RFP production (Y- axis) using the IncuCyte live cell image system. (PDF) [file ppat.1009577.s008.pdf]

S8 Fig

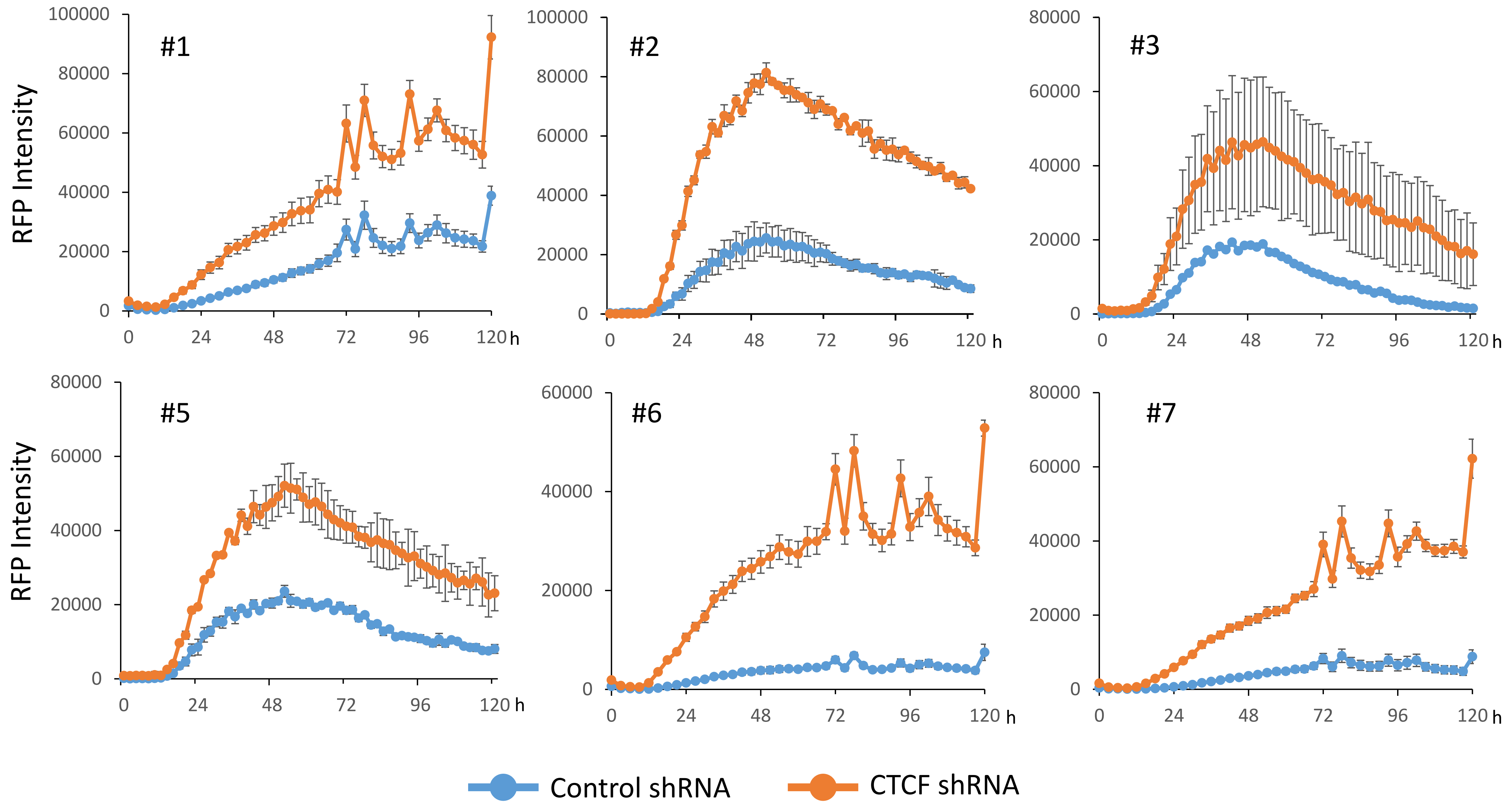

RFP Intensity
#1
#2
#3
#5
#6
#7
100000
80000
60000
40000
20000
0
0
24
48
72
96
120 h
Control shRNA
CTCF shRNA

S8 Fig

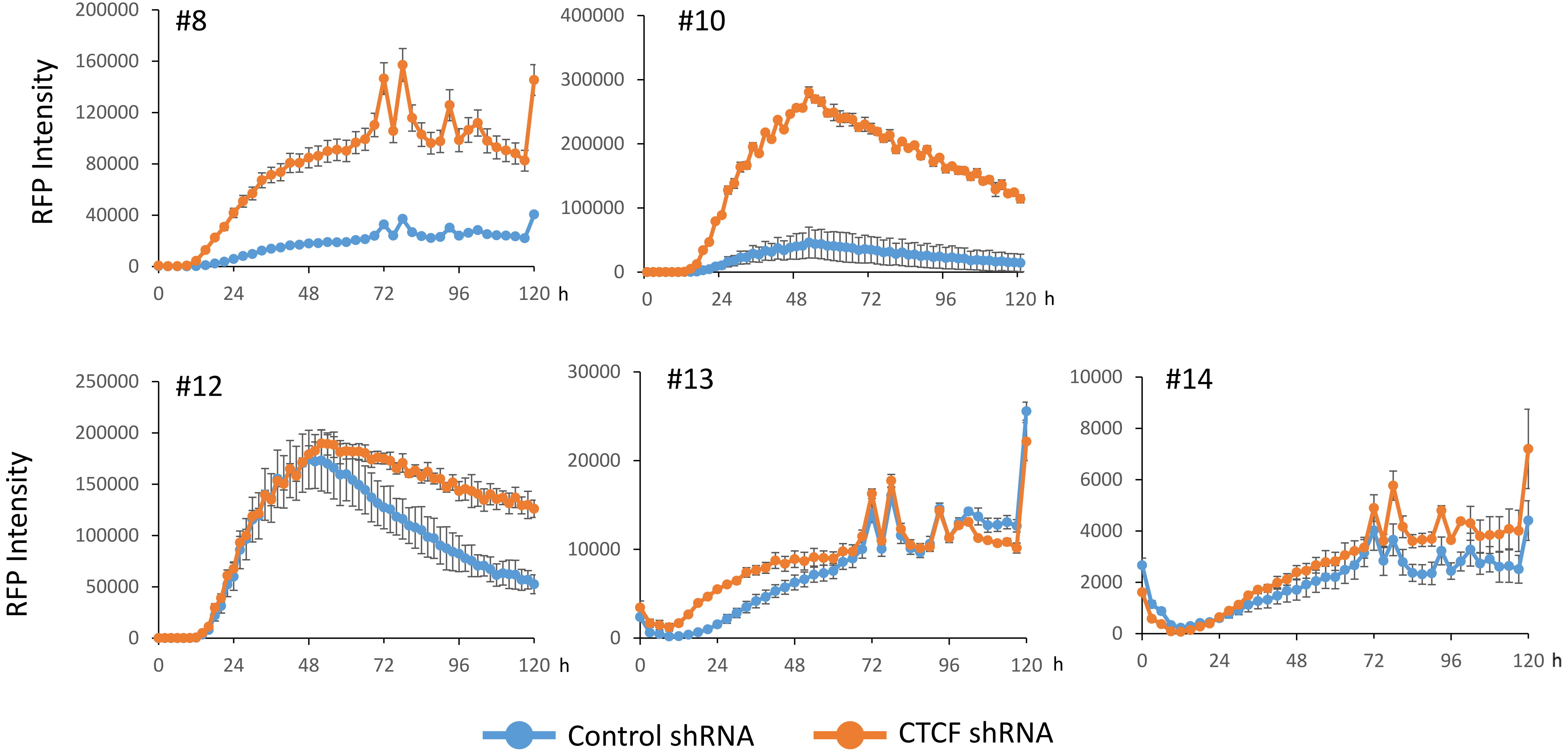

S8 Fig. Inhibition of CTCF binding to HTLV-1 provirus affects proviral gene transcription

Clonal HTLV-1 infected JET cell lines were generated, each carrying a single, latent provi**o**irus at a unique integration site. Viral gene expression was activated with PMA/Ionomycin and monitored by measuring Tax mediated RFP production (Y- axis) using the IncuCyte live cell image system.
