# Supplementary material for: Epigenomic regulation of human T-cell leukemia virus by chromatin-insulator CTCF
Source: PLoS Pathog. 2021 May 21;17(5):e1009577. doi: 10.1371/journal.ppat.1009577 (PMC8174705; doi:10.1371/journal.ppat.1009577)
Supplement: S9 Fig — Clonal HTLV-1 infected JET cell lines were generated, each carrying a single, latent provirus at a unique integration site. Viral gene expression was activated with CD3/CD8 antibodies and monitored by measuring Tax mediated RFP production (Y-axis) using the IncuCyte live cell image system. (PDF) [file ppat.1009577.s009.pdf]

S9 Fig

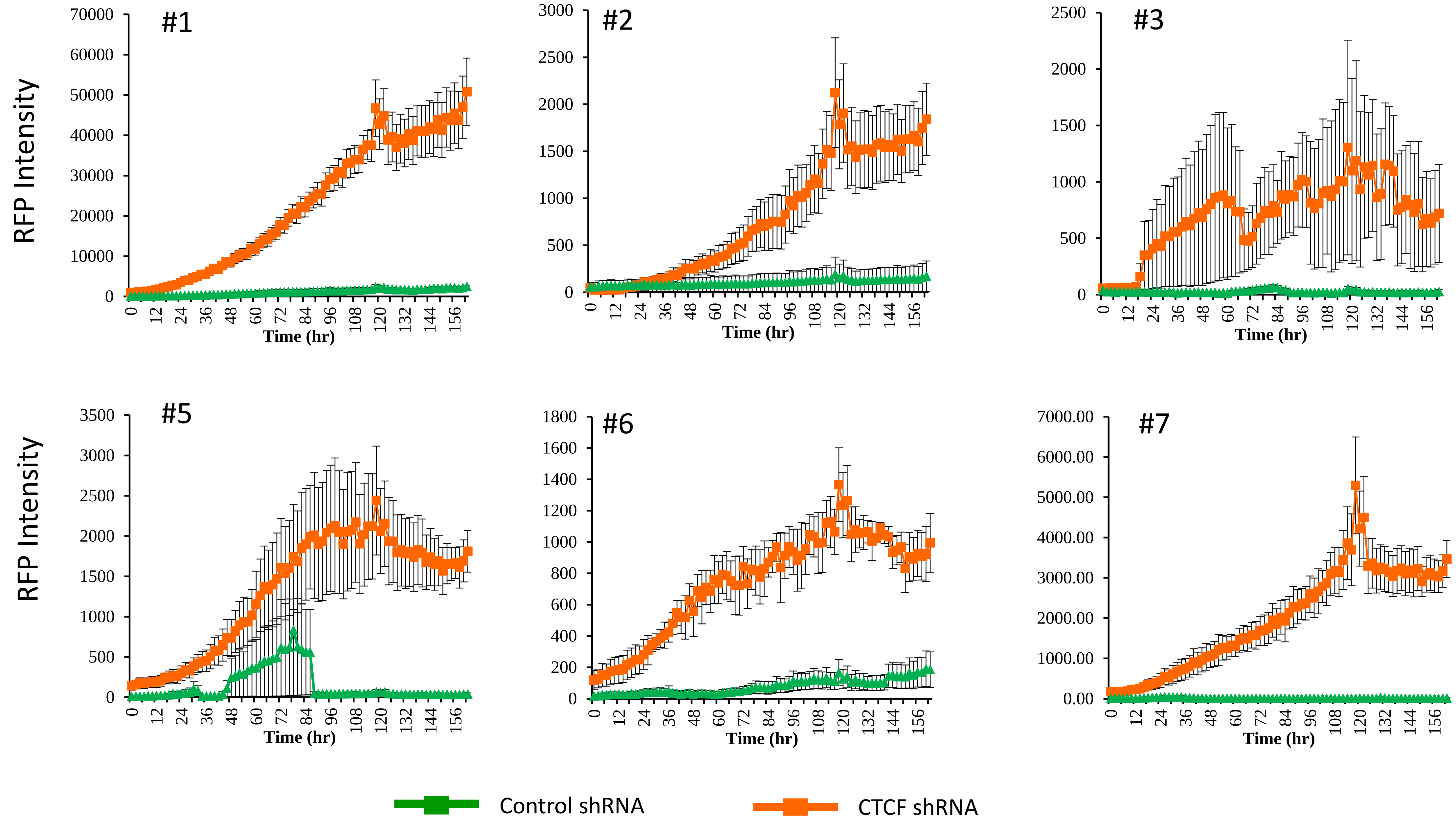

#1
#2
#3
#5
#6
#7
RFP Intensity
RFP Intensity
Time (hr)
Time (hr)
Time (hr)
Time (hr)
Time (hr)
Time (hr)
Control shRNA
CTCF shRNA

S9 Fig

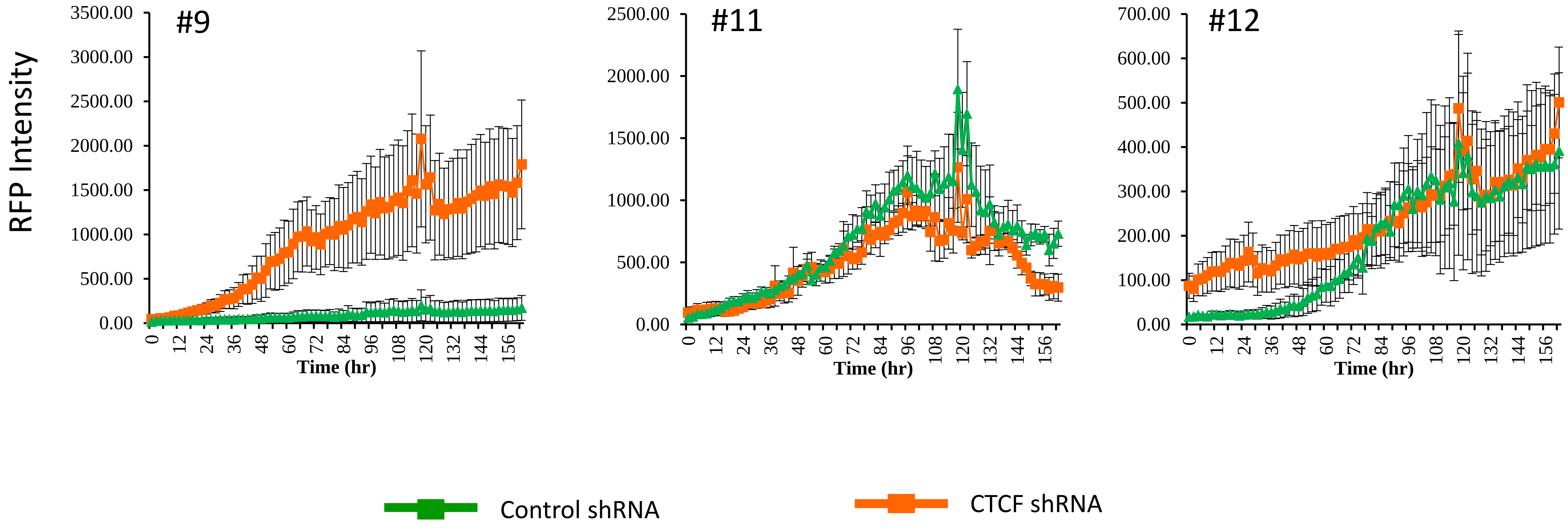

S9 Fig. Inhibition of CTCF binding to HTLV-1 provirus affects proviral gene transcription

Clonal HTLV-1 infected JET cell lines were generated, each carrying a single, latent provirus at a unique integration site. Viral gene expression was activated withCD3/CD8 antibodies and monitored by measuring Tax mediated RFP production (Y- axis) using the IncuCyte live cell image system.
