# Supplementary material for: Epigenomic regulation of human T-cell leukemia virus by chromatin-insulator CTCF
Source: PLoS Pathog. 2021 May 21;17(5):e1009577. doi: 10.1371/journal.ppat.1009577 (PMC8174705; doi:10.1371/journal.ppat.1009577)
Supplement: S12 Fig — DNA methylation of the HTLV-1 provirus is presented as the percentage of methylated CpG (Y- axis) at the indicated locations of the viral DNA in pX region (X-axis). The schematic diagram of HTLV-1 provirus indicates the regions examined by bisulfite treatment and DNA sequencing as described in the Materials and Methods. The number of each clonal cell line is labeled on the right side of the figure. (PDF) [file ppat.1009577.s012.pdf]

S12 Fig

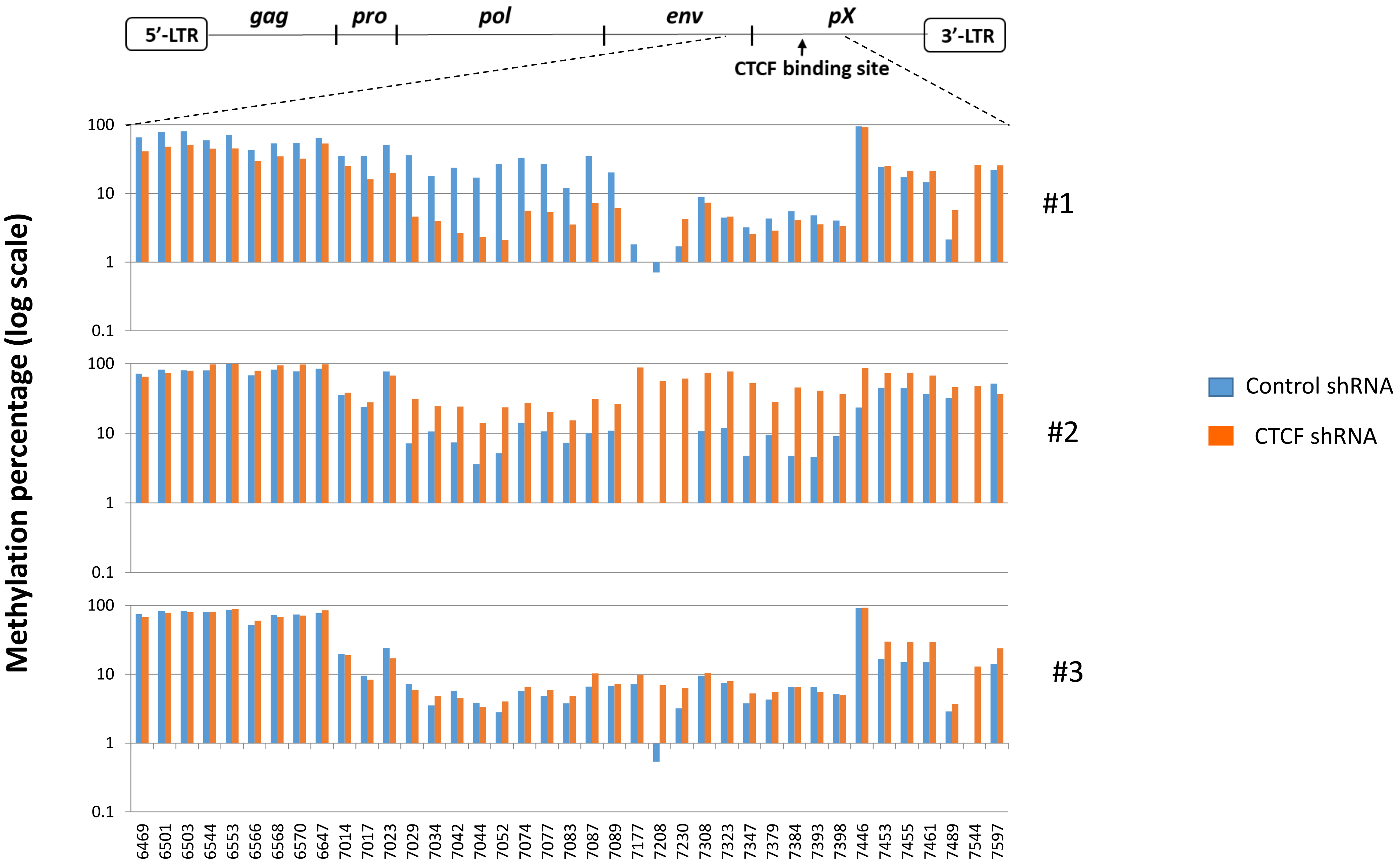
5'-LTR
gag
pro
pol
env
pX
3'-LTR
CTCF binding site
Methylation percentage (log scale)
100
10
1
0.1
#1
#2
#3
Control shRNA
CTCF shRNA
6469
6501
6503
6544
6553
6566
6568
6570
6647
7014
7017
7023
7029
7034
7042
7044
7052
7074
7077
7083
7087
7089
7177
7208
7230
7308
7323
7347
7379
7384
7393
7398
7446
7453
7455
7461
7489
7544
7597

S12 Fig

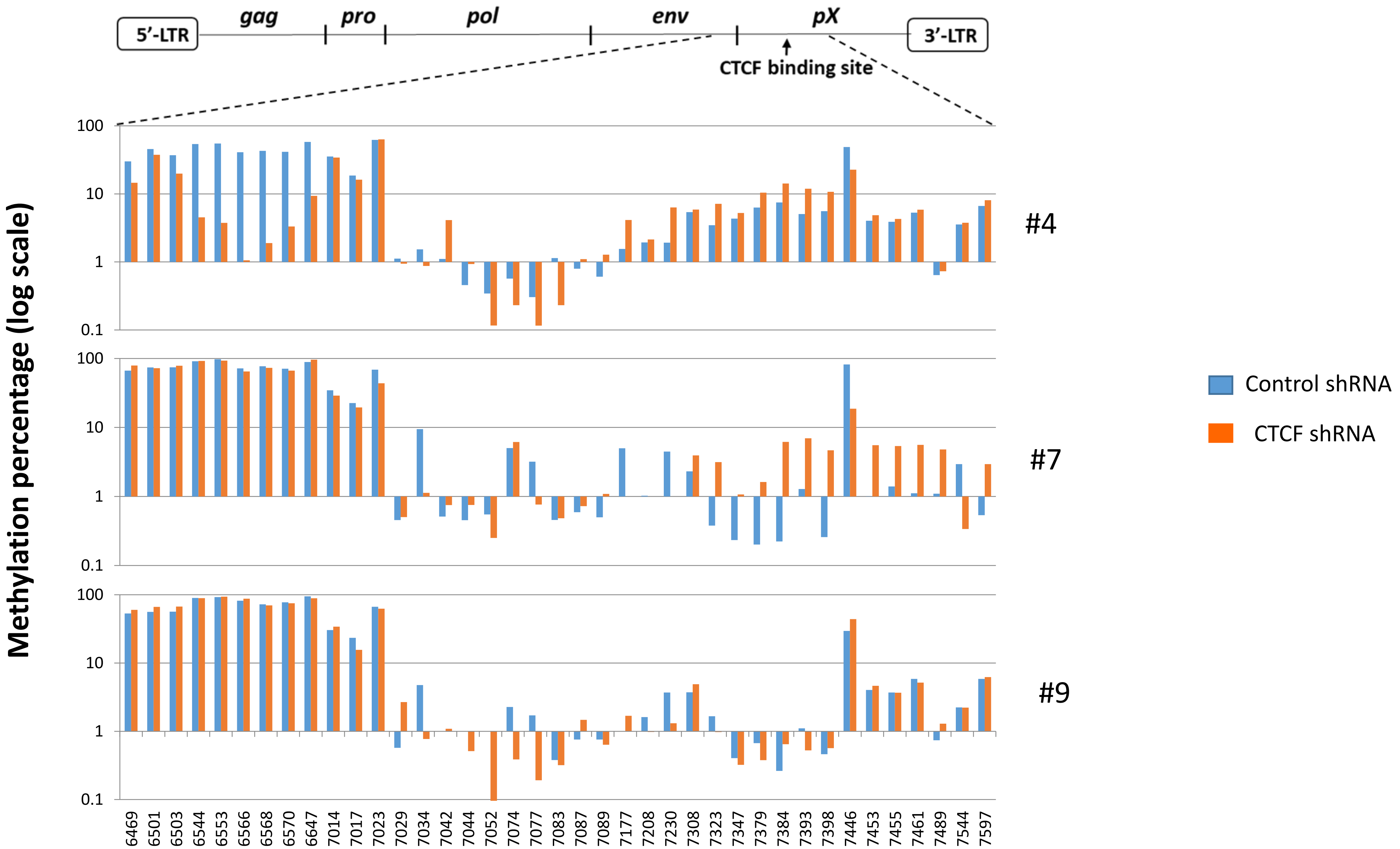

5'-LTR
gag
pro
pol
env
pX
3'-LTR
CTCF binding site
Methylation percentage (log scale)
100
10
1
0.1
#4
#7
#9
Control shRNA
CTCF shRNA
6469
6501
6503
6544
6553
6566
6568
6570
6647
7014
7017
7023
7029
7034
7042
7044
7052
7074
7077
7083
7087
7089
7177
7208
7230
7308
7323
7347
7379
7384
7393
7398
7446
7453
7455
7461
7489
7544
7597

S12 Fig

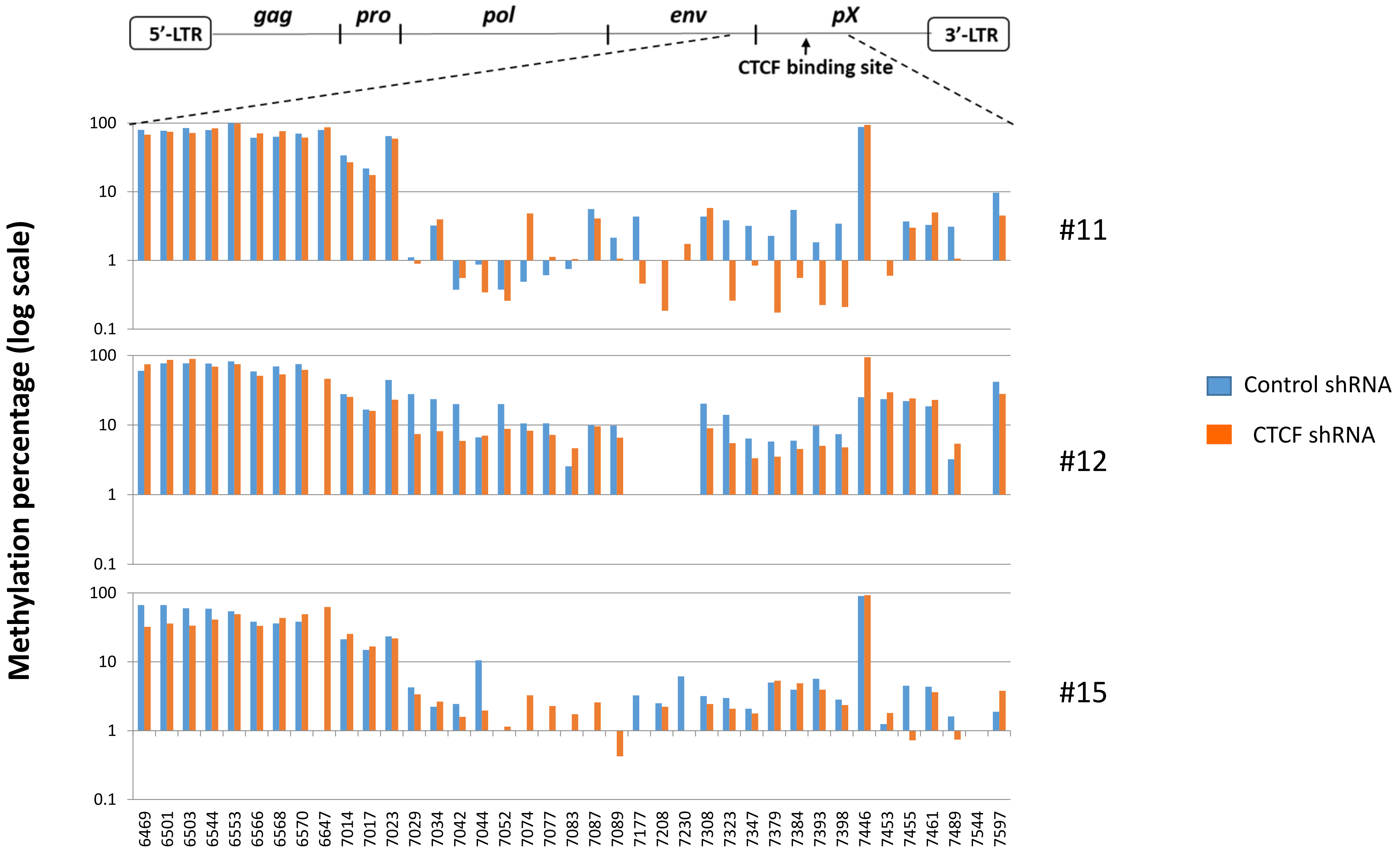
5'-LTR
gag
pro
pol
env
pX
3'-LTR
CTCF binding site
Methylation percentage (log scale)
100
10
1
0.1
#11
#12
#15
Control shRNA
CTCF shRNA
6469 6501 6503 6544 6553 6566 6568 6570 6647 7014 7017 7023 7029 7034 7042 7044 7052 7074 7077 7083 7087 7089 7177 7208 7230 7308 7323 7347 7379 7384 7393 7398 7446 7453 7455 7461 7489 7544 7597

S12 Fig. CTCF knock down by shRNA in JET clonal cell lines results in expansion of DNA methylation in the pX region of the provirus

DNA methylation of the HTLV-1 provirus is presented the percentage of methylated CpG (Y- axis) at the indicated locations of the viral DNA in pX region (X-axis). The schematic diagram of HTLV-1 provirus indicates the regions examined by bisulfite treatment and DNA sequencing as described in the Materials and Methods. The number of each clonal cell line is labeled on the right side of the figure.
