# Supplementary material for: Epigenomic regulation of human T-cell leukemia virus by chromatin-insulator CTCF
Source: PLoS Pathog. 2021 May 21;17(5):e1009577. doi: 10.1371/journal.ppat.1009577 (PMC8174705; doi:10.1371/journal.ppat.1009577)
Supplement: Attachment — Submitted filename: Response to Reviews10APR2021.docx [file ppat.1009577.s017.docx]

Responses below to Reviewer Comments enumerated below.

Line numbers refer to version of manuscript with no track changes

Summary of Changes Made in Response to Reviews:

- New Fig 6 and Fig S9 with methylation alterations in clonal JET cell lines
- Moved previous Fig S10 to Fig 7 concerning latency studies
- Added Fig S8 with response to anti-CD3 and anti-CD28
- Added Table S1 describing CD4 and CD8 proportions in immortalized PBMCs
- Added Table S2 describing viral gene expression in immortalized PBMCs
- Expanded Discussion concerning breakdown of epigenetic barriers
- Expanded Discussion concerning differences from Miura et al paper
- Expanded Discussion regarding limitation of the study
- Provided additional information or corrections concerning other minor critiques

Reviewer Comments (if any, and for reference):

Reviewer's Responses to Questions

**Part I - Summary**
Please use this section to discuss strengths/weaknesses of study, novelty/significance, general execution and scholarship.

Reviewer #1: In this manuscript, Cheng et al. investigate potential functions of the CTCF binding site in the HTLV-1 genome. The authors generated a CTCF mutant proviral clone that disrupts CTCF binding, which was then used to infect PBMCs and JET cells. Since the CTCF mutations also result in p12 mutations, the authors introduced a stop codon in p12 upstream of the CTCF mutations. A proviral clone with only the p12 deletion was used as a control. The authors found increased DNA methylation in the genomes of delta CTCF viruses in both PBMCs and JET cells, mainly downstream of the CTCF binding site. There was also an increase in both activating (H3K4me3) and repressive (H3K27me3) histone marks in delta CTCF viruses in PBMCs and JET cells. Finally, mutation of the CTCF binding site did not impact proviral integration site preferences, but did influence proviral gene expression. Knockdown of CTCF with shRNA enhanced viral gene expression in JET cells infected with wild type HTLV-1, but not delta CTCF viruses.

Overall, the results suggest that the CTCF binding site serves as an epigenetic barrier to limit the spread of methylation to the 3’ LTR and thus protect HBZ from inactivation, however it is difficult to understand why there were no noted changes in HBZ expression associated with loss of the CTCF binding site. Furthermore, the results from this study are directly opposed to those from a recent study (Miura et al. 2018 Wellcome Open Research 3:105), and thus additional experiments are warranted to rigorously substantiate the conclusions. The presented data convincingly implicate CTCF in the suppression of plus strand HTLV-1 gene expression; however, it remains unclear if the underlying mechanism is due to epigenetic changes (i.e. DNA and histone methylation) in the HTLV-1 genome caused by loss of the CTCF binding site.

Response 1 – In the Discussion (lines 431-435) we added:

“However, in the current study, we did not detect any significant reduction of *hbz* mRNA in our cell clones which showed increased expression of Tax protein when CTCF was knocked down. One explanation could be that the *hbz* mRNA level may not accurately reflect the level of HBZ protein, which inhibits Tax expression by competing for binding of the transcription co-factor CREB.”

Reviewer #2: Recent work demonstrated the existence of a CTCF binding site in the HTLV-1 provirus (Satou, PNAS). This is a nice paper reporting the effects on DNA methylation and expression of HTLV-1 proviral DNAs, upon mutating the CTCF binding site. The usual switch from highly methylated upstream (in the “sense” direction), to poorly methylated downstream, is mostly lost in the mutant. This is a very intriguing result (predicted by early studies and here confirmed to be true). Histone marks were also affected.
All the work here is done with cell lines infected in vitro, so we are looking at integration sites established by recent infection. The work was done in pooled JET cells and immortalized clones of PBMC cells.

The authors next looked effects of KD of CTCF on transcription, using infected JET clones that are initially silent, and then activated with PMA and ionomycin, plus/minus KD of CTCF. The KD gave increased viral gene expression in a subset of the clones (not all).
There was no effect of the mutation on the frequency of establishment of latently infected clones or the reactivation from latency. Similar results to this were recently reported by some of the authors (and are cited here: Martinez, Retrovirology). There were no changes in integration site preferences.

Somewhat conflicting results on the effects of CTCF BS mutation on viral chromatin were recently reported (Wellcome Open Research. 2018;3:105) in a different setting.

Response 2 – We expanded the Discussion of differences from the Wellcome Open Reseach 2018 manuscript with the following on lines 474-494 (with the changes from the original manuscript shown in italics):

“But Miura et al later reported that removal of the vCTCF-BS did not affect the epigenetic modifications and transcriptional activity of the provirus in two HTLV-1 patient-derived T-cell clones, each carrying one copy of the provirus lacking the vCTCF-BS [17]. *Their methylation analysis was based on ten or fewer clones in each experiment*. In order to more completely evaluate the role of CTCF binding, we established multiple HTLV-1 infected cell clones, each carrying a single copy of the provirus integrated at a unique chromosomal location. *In addition, we used next generation sequence methods to analyze several thousand reads for each potential CpG site.* By examining these infected cell lines, we were able to investigate the effect of the CTCF binding on reactivation of the provirus in various genomic environments of the host. Notably, in the majority of clonal cell lines, decreased levels of CTCF led to increased methylation in pX region and tax gene expression, demonstrating that the CTCF binding plays a critical part in the regulation of viral DNA methylation and gene expression in the majority of cell clones infected with WT HTLV-1. *However, because the actions of CTCF at a given location depend on the local context, involved in site-specific interactions between CTCF, transcriptional cofactors, and the primary DNA sequence (for example, enhancers and silencers), removal of CTCF does not invariably lead to changes in chromatin looping and epigenetic marks flanking the CTCF binding site [58, 59].* Therefore, differences in proviral integration sites may explain why one-third of our clonal lines and two of Miura’s were unaffected by CTCF knockdown, and also why ablation of the vCTCF-BS did not result in methylation increase in all CpG sites of the pX region in the infected PBMCs and JET cells, each of which contain about 3000 unique integration sites.“

This is a generally nice story on an interesting site in the HTLV-1 genome. There’s a lot of work presented, and the findings are generally convincing and properly interpreted. There are limitations to the settings (cells, timing) that are not ideal but unavoidable, and just should be admitted firmly somewhere in discussion.

Response 3 – We added the following to the Discussion on lines 495-498

“Some limitations of our current work are the use of a single virus with a mutation in the vCTCF-BS, studies in restricted to Jurkat cells and primary human lymphocytes, lack of dynamic continuous monitoring of *hbz* expression in JET cell lines, and use of a surrogate for continuous monitoring of *tax* expression based on RFP expression.”

Reviewer #3: In this manuscript, Cheng and Joseph et al. generated and examined an HTLV-1 mutant virus that eliminates the binding site for the epigenomic insulator CCCTC-binding protein (CTCF) located in the pX region of the viral genome. Using a variety of state-of-the-art techniques and clonally infected T-cells, the authors clearly show that mutation of the CTCF-binding site (CTCF-BS) does not disrupt viral gene expression. Moreover, establishment of latency and reactivation from latency do not seem to depend on the CTCF-BS. However, DNA deep sequencing of bisulfite treated DNA revealed that the viral genome is heavily methylated upstream of the CTCF-BS, while it is only weakly methylated downstream of the CTCF-BS. Interestingly, disruption of the CTCF-BS leads to a dramatically enhanced methylation in the region downstream of the CTCF-BS. ChIP sequencing data underline the insulating role of the viral CTCF-BS since disruption of CTCF-BS results in differentially increased histone methylation of the HTLV-1 provirus. Using next generation sequencing, the authors show that disruption of the CTCF-B does not impact proviral integration site preferences of HTLV-1. However, analysis of a series of clonally infected cells by qPCR and live cell imaging revealed that inhibition of CTCF binding affects viral gene expression in ca. 2/3 of all investigated clones, leading to increased viral gene expression. Finally, the authors show that cells infected with delta-CTCF-BS virus do not differ in establishment of viral latency nor do they differ in viral reactivation from latency, suggesting that control of latency may be independent of CTCF-binding.
This is a very interesting manuscript which clearly elucidates the relevance of the CTCF-BS in HTLV-1 proviral DNA using a broad panel of up-to-date methods. The manuscript is well-written and the experiments are clearly described. The authors comprehensively analyzed the newly established proviral clones and the respective controls. The experimental results strongly support the conclusions of the authors. Due to the importance of the data showing that mutation of the CTCF-BS does not impact latency establishment and reactivation (Fig S10), these data should be shown in the main part of the manuscript.

Response 4 – The previous Fig S10 is now part of the main manuscript and shown as Fig 7

**Part II – Major Issues: Key Experiments Required for Acceptance**
Please use this section to detail the key new experiments or modifications of existing experiments that should be absolutely required to validate study conclusions.

Generally, there should be no more than 3 such required experiments or major modifications for a "Major Revision" recommendation. If more than 3 experiments are necessary to validate the study conclusions, then you are encouraged to recommend "Reject".

Reviewer #1: 1) The authors need to characterize the HTLV-1 immortalized PBMCs used in the study. Are these cells CD4+, IL-2 dependent, etc.? What is the baseline expression of Tax, HBZ and Gag mRNA and protein in PBMCs immortalized with WT, p12Stop and delta CTCF viruses? Based on the authors’ model, differences in viral gene expression should be apparent in the absence of a functional CTCF binding site in the viral genome.

Response 5 – These data are added to the Results section (lines 178-183) and Tables S1 and S2. The description in the Results section is as follows:

“Multiple different IL2-dependent PBMC-derived cell lines had varying proportions of single or double positive or double negative CD4+ and CD8+ cells (Table S1). However, no significant differences were seen in comparison of PBMCs infected with HTLV-1ΔCTCF or HTLV-1 or HTLV-1 p12Stop. Levels of *tax* and *hbz* RNA and production of p19 capsid protein per cell were slightly higher in PBMCs infected with HTLV ΔCTCF than those infected with WT HTLV-1 or HTLV-1 p12Stop (Table S2).”

2) It is unclear if the methylation studies presented in Figs. 2 and 3 represent single cell clones or the bulk population of PBMCs and JET cells. This needs to be clarified by the authors. If these experiments were performed with the bulk population of cells, they should be repeated with single cell clones with defined viral integration sites. Why is the overall histone methylation so low in JET cells (Fig. 3)?

Response 6 – We clarified that the data in Fig 2 and 3 are from bulk cells on lines 184-185 of the Results:

“In order to analyze DNA methylation, we used next generation sequence analysis of bisulfite-treated proviral DNA from the bulk populations of JET cells and PBMCs”

And in the Fig 2 legend (lines 964-965):

Upper panel: HTLV-1 immortalized bulk population of PBMCs; lower panel: HTLV-1 infected bulk population of JET cells.”

And in the Fig 3 legend (lines 971-973):

“Plots show fold enrichment over input for the indicated chromatin modifications on HTLV-1 provirus DNA in the bulk populations of JET cells (left) or PBMCs (right).

Concerning the low overall histone methylation in JET cells, we added the following to the Results on lines 240-242 as well as Figure S6.

“Lower levels of histone methylation marks, normalized to input, were found in JET cells compared to PBMCs, likely due to shorter duration of infection. The same data are shown in Figure S6, without normalization for input values.”

Response 7 – We added data in the new Figure 6 showing DNA methylation data in 6 randomly selected type I cloned infected JET cell lines and 3 randomly selected type II cloned infected JET cell lines. The description of these experiments are in the Results on lines 301-306:

“We also examined methylation of the viral DNA around the vCTCF-BS in these clonal lines using next generation sequence analysis of bisulfite-treated proviral DNA. The CTCF knockdown induced a significant increase of methylation in most type I but not type II cell lines (Fig 6). Four type I lines (67%) exhibited a higher average fold increase than type II lines. Therefore, elevated Tax expression in the majority of type I cell lines positively correlates with enhanced methylation in the pX region when CTCF is down regulated.”

Response 8 – Concerning the lower level of DNA methylation in the HTLV-1ΔCTCF provirus in JET cells compared to PBMCs, we added the following to the Results on lines 196-197

“It is unclear whether this is a result of cell type differences, or differences in the duration of infection.”

3) The data in Fig. 5 suggest that CTCF binding to the proviral genome suppresses Tax expression. However, what is the molecular basis of the increased plus strand viral gene expression in these particular JET cell clones? Is it due to epigenetic changes? Is there increased H3K4me3 marks at the 5’ LTR? Do viral integration sites play a role? Why is HBZ expression unaltered in the different clones?

Response 9 – Concerning HBZ expression, see Response 1. Our CHIPseq methodology did not distinguish the 5’ and 3’ LTRs. We discussed the molecular basis for increased plus strand expression in the Discussion on lines 391-420.

“In other contexts, CTCF loss mediates unique DNA hypermethylation surrounding CTCF binding sites in a variety of human cancers [38]. In the prostate, the Igf2-H19 locus experiences DNA methylation with CTCF downregulation at multiple intergenic CTCF sites [39]. A study of seven immortalized cell lines and twelve normal cell cell types showed that DNA methylation was linked to CTCF binding at 40% of sites across the genome [40]. It was suggested that variation in CTCF occupancy may derive from a complex regulation of specific interaction partners [41]. Others suggested that loss of CTCF expression might activate DNA methyl transferases [42]. However, we found no correlation of changes in HTLV-1 gene expression in response to demethylating drugs with the phenotypes identified in our type I and type II infected JET cell lines (Figure 5 and not shown). Alternatively, DNA methylation may prevent CTCF binding [43, 44]. The correlation between methylation site results and positive strand gene expression (Figures 5 and 6) suggests a significant relationship between these findings. However, additional studies are required to identify the precise mechanisms involved.

Whole-genome studies have shown that exons are more highly methylated than introns and transitions in the degree of methylation occur at exon-intron boundaries, possibly suggesting a role for methylation in regulating splicing [45]. Shukla et al demonstrated that CTCF binding results in pausing of RNA pol II and changes in the kinetics of Pol II movement, thus influencing splicing [46]. They further showed that whether exons are favorably included or not in mature transcripts depends on the position of CTCF binding sites. Interestingly Satou et al recently reported that CTCF down regulation by shRNA leads to reduced p30 expression and postulated that CTCF plays a role in the regulation of HTLV-1 RNA splicing because the vCTCF-BS is near the splice junction of the p30 transcript [15]. In the current study, we observed a significant increase of proviral methylation in the pX region, and Tax expression in two thirds of our clonal cell lines when CTCF is knocked down. Intriguingly the vCTCF-BS is located immediately upstream of the third exon of the *tax/rex* transcript, thus RNA splicing could be a step where CTCF exerts its impact. However, the exact mechanism of how CTCF binding influences processing of viral transcripts remains to be elucidated. In addition, Satou’s report also revealed that some sequences near the vCTCF-BS exert CTCF-dependent enhancer blocking activity, thus loss of the vCTCF-BS could relieve the block which could consequently promote transcription driven by the 5’ or 3’LTR if an enhancer is involved in expression of either proviral strand.”

4) PMA/Ionomycin is not a physiologically relevant approach to induce viral gene expression (Fig. 5). Crosslinking of anti-CD3 and anti-CD28 with agonistic antibodies more accurately mimics T cell activation. Recent studies (Kulkarni et al. 2017 Cell Chem. Biol. 24: 1377-87; Kulkarni et al. 2018 JCI Insight 3: e123196; Mahgoub et al. 2018 PNAS 115: E1269-E1278) have identified stress stimuli as transcriptional activators of the HTLV-1 plus strand. Do stress stimuli (e.g. hypoxia or oxidative stress) also reactivate HTLV-1 gene expression in the latent JET cell clones?

Response 10 – We did not examine other stress stimuli. We did repeat gene expression studies with anti-CD3 plus anti-CD28. These experimental data are shown in the new Fig S9, and described in the Results on lines 298-300.

“To test if the viral gene expression can be reactivated through a more natural pathway, we treated cells with CD3/CD28 antibodies. As expected, similar results were observed except that CD3/CD28 stimulation was much weaker (Figure S9).”

**Reviewer #2**: Some issues:
(ll 226 ff.):It would have been nice to have looked at one more silencing mark, which is H3K9me3.

Response 11 – We did not examine H3K9me3.

ll 345 ff: The discussion of the border phenomenon is important, but should really start out with an explanation of the fact that we do not yet know the point of origin of the methylation area, and that this would be important to identify. Then the discussion of where it might be is more clear. (Also we need some statement of the premise that it can spread.) I don’t think the data really rule out the idea that the 5’LTR might be the source of the methylation domain, though Taniguchi may be right.

Response 12 – We expanded the Discussion on lines 362-390 concerning the premise for spread of methylation, with additions shown in italics below, as well as the points mentioned in Response 9:

“In the current study, we showed that elimination of the vCTCF-BS not only causes remarkable enhancement of methylation in the pX region, shifting the border of this epigenetic modification closer to the 3’-LTR, but also significantly increases the methylation on the antisense strand encoding HBZ. It was postulated that by forming a barrier to epigenetic modifications, CTCF may cause or perpetuate differential transcription of the 5′ and 3′ parts of the provirus, resulting in persistent expression of HBZ, which is required for clonal persistence [13]. Although how methylation of the proviral DNA is regulated is still unknown, and the precise mechanism of how methylation controls gene expression of the provirus is also not well understood, it is clearly established that the 5’-LTR plays a key part in controlling sense strand transcription [31]. Based on the observation that the *gag, pol* and *env* sequences of the provirus are heavily methylated, Tanigichi et al speculated that DNA methylation first occurs in the *gag, pol* and *env* regions and then extends in the 5’ and 3’ directions *in vivo*; when the 5’-LTR becomes methylated, viral transcription is silenced [37]. Miura et al. later showed that the plus strand expression was silenced even though the 5’LTR was hardly methylated, implying that DNA methylation at the 5’LTR is not the sole factor that suppresses the viral transcription [17]. Intriguingly they further demonstrated that the pattern of hypermethylation in internal sequences of provirus in various cell lines did not change regardless of the sense strand expression. This finding indicated that the methylation pattern of the internal DNA is an innate characteristic of the provirus and independent of integration location. However, we cannot exclude the possibility that expanded methylation may exist in some infected cells, but not be detected due to the limitations of the methods utilized. It is possible that suppression of sense strand transcription requires methylation of the 5’-LTR in conjunction with hypermethylation of the internal sequences of the provirus. Thus, extending methylation beyond the established border in the pX region could potentially influence the regulatory function of the 5’-LTR for sense strand transcription. However, the final effect of the extended hypermethylation in the pX region likely depends on a combination of methylation status in both the internal and LTR regions. Interestingly increased methylation in the 5’ and 3’-LTR was also detected in the HTLV-1ΔCTCF provirus but at lower levels, which seems to agree with the speculation of Taniguchi and colleagues that methylation starts in the internal sequences then extends to the 5’ and 3’ LTR [37].”

ll 244: While the DNA methylation looks like a border issue, the histone modification does not. The summary statement that “loss of the CTCF binding site in HTLV-1 results in increased histone methylation 5’ and 3’ of the CTCF site, suggesting that CTCF binding may act as a boundary element to epigenetic modification” does not quite make sense. This does not seem like a boundary element. Since the effect is the same on both sides, it just seems like a site working in both directions.

Response 13 – We added to the Discussion (lines 445-465) points addressing this issue:

“Interestingly both active (H3K4Me3, H3K36Me3) and repressive (H3K27Me3) histone marks were enhanced in HTLV-1ΔCTCF infected cells. Although H3K27Me3 is known to be a characteristic of silencers, unlike H3K9me3 which remains silenced all the time and prevents multiple transcription factors (TFs) from binding, H3K27me3 still allows certain genes to be activated through TF binding in a different cell state [49]. Furthermore, this function is cell type specific and highly context dependent. It can act as a silencer in one cell line but a super enhancer in another cell line [50-56]. Thus it is not a determinant factor of silencers on its own [57]. It has been shown that H3K27Me3 and H3K4Me3 frequently coexist in genes expressed at low levels in embryonic stem cells (ES), and it was proposed that bivalent domains silence developmental genes in ES cells while keeping them poised for activation [49]. It is likely that viral gene expression is also regulated simultaneously by both active and repressive histone marks. The opposite effect of these two functions could enable a fine-tuning of the viral gene expression and provide great sensitivity and robustness to the regulatory circuitry of plus and minus strand expression, especially for *tax* and *hbz* transcripts.

Surprisingly we only observed a clear border for H3K4me3 modification in HTLV-1 infected JET cells but not the other histone methylation marks. Significant increases of both H3K4Me3 and H3K36Me3 due to the vCTCF-BS deletion were also detected on both sides of the vCTCF-BS. Therefore the role of CTCF as a boundary element for histone methylation may not be as important as for DNA methylation. Both DNA methylation and histone modification have been reported to affect gene expression of the HTLV-1 provirus [17, 23, 37]. Future studies should investigate how these two mechanisms interplay to collectively regulate proviral gene expression.”

It would be nice to have more discussion of the magnitude of the various effects. For example, Fig 2 is impressive. But Fig 5 (expression) is showing generally more modest effects (2-fold or so, often). No point hiding this.

Response 14 – The new data in Fig 6 show that the magnitude of changes in DNA methylation in individual cell lines are variable.

The results of the Bangham paper probably need some more discussion, including how the setting is different than here.

Response 15 – See Response 2

Reviewer #3: No additonal experiments required, but Fig S10 should be presented in the main manuscript and not in the supplement.

Response 16 – See Response 4

**Part III – Minor Issues: Editorial and Data Presentation Modifications**
Please use this section for editorial suggestions as well as relatively minor modifications of existing data that would enhance clarity.

Reviewer #1: 1) JET cells should be defined when first introduced in the Results section. It is not common knowledge that these cells express tdTomato controlled by Tax responsive elements.

Response 17 – A sentence describing JET cells was added in the Results on lines 177-178

“JET cells carry a tdTomato red fluorescent protein (RFP) under the control of a Tax responsive element.”

Reviewer #2: Small points:
The reportage of the changes in CpG methylation up- and down-stream of the CTCF BS in the text is someone long-winded and unnecessarily detailed. The numbers are appropriately in the table, and a more general description would be better. Summary (ll. 180 area) is good.

And the format of the fold changes given as “2-31 fold” is somewhat confusing – maybe it needs to be written as “in a range of 2-fold to 31-fold” or something similar. But we don’t really need all the numbers in the text.

Response 18 – We shortened the description of changes in CpG methylation up- and downstream of the vCTCF-BS in the Results on lines 184-212.

(ll. 192 ff) Scoring the 5’LTR vs. 3’LTR for methylation needs explanation – if the assay is by PCR after bisulfite, we need to know how the reads are assigned to either one or the other LTR. (Maybe the reads are long enough to discriminate?)

(ll 199 ff) We need a note as to how strand-specific methylation is read out – presumably seeing C-to-T changes on the appropriate strand (i.e. CpG to TpG as opposed to seeing CpG to CpA change).

Response 19 – The methods for distinguishing the 5’LTR vs 3’LTR for methylation were provided on lines 571-575:

“For the LTR region, two step PCR was performed in order to separate 5’ LTR and 3’LTR region. To amplify 5’ LTR, a BSP forward primer was designed in the start of the 5’U3 LTR region and a BSP reverse primer was designed in the start of gag region. For the antisense 3’LTR region forward primer was located in the HBZ region whereas the reverse primer was in the U5 portion of the 3’ LTR region.”

(ll 240 ff) It needs to be stated here that (very curiously) both repressive and activating histone marks are increased In the mutant. This is hard to interpret, but it should be laid out here.

Response 20 – See Response 13

Reviewer #3: Line 127: Electrophoretic mobility shift data is not shown. Either show it in the supplement or do not mention it.

Response 21 – The EMSA is shown in the manuscript by Martinez MP et al (reference 18)

Line 174: please cite studies that „commonly used“ JET cells.

Response 22 – References 25-27 were provided in the Results section on line 175

Line 238: typo, HTLV-1A should read HTLV-1

Response 23 - Corrected

Line 254: please provide a reference for the Gini index.

Response 24 – Provided as Reference 30

Figures:
All Figures should be formatted according to the journal guidelines. Some figures are too wide.

Response 25 – Figures were formatted according to journal guidelines

Fig.2: The left and the right part of the Figure should be adjusted to each other, or the Figure should be arranged in upright format. It would be helpful for the reader if the authors create subpanels, e.g. A) percentage of methylation, and B) change of methylation.

Response 26 – The left and right part of Fig 2 were adjusted to one another. We prefer the landscape format. Subpanels A and B were provided

Fig.4A: Why did the authors not analyze HTLV-1 wildtype? Since integration sites of HTLV-1-Delta-CTCF were analyzed, the legend should also read „Delta-CTCF“ instead of CTCF.

Response 27 – A sentence was added to Fig 4 legend on lines 982

“Inconsistent results were obtained with HTLV-1 WT infected cells.”

Fig. 4B: It is not clear why the authors show the inset. Labeling of the axes is lacking in the inset. The authors state FOXP3 in the legend, but not in the text.

Response 28 – Fig 4B is corrected with labeling of the axes. We do not consider it necessary to state FOXP3 in the Results section.

Fig S3: The labeling of the luciferase assay and the western blot should be the same (WT=p12wt, Delta-CTCF=p12tr), otherwise, it is confusing for the reader.

Response 29 – Fig S3 labeling has been corrected as suggested

Fig. S4: Copy numbers of HBZ are very low (0.008 per million GAPDH). Could the authors please comment if these low copy numbers are typical for HBZ expression analysis of proviral clones?

Response 30 – A sentence was added in the Results on lines 297-298

“Levels of HBZ mRNA are typically 10^3^ – 10^4^ lower than that of Tax and Gag-Pol mRNA [32-34].”

Fig. S5: The labeling of subpanels in the legend is mixed up. It should read „in (A) 5’LTR, (D) 3’LTR and HBZ“ and „in (B) PBMCs and (C) JET“; Typo: THLV-1; * and ** should also be explained in the legend

Response 31 – Fig S5 legend corrected

Fig. S7: Why is #15 shown twice? (in Fig. 5B and in FigS7)

Response 32 – Fig S8 was corrected (as #14)

Line 549: Typo „).“

Response 33 – Typo corrected

PLOS authors have the option to publish the peer review history of their article ([what does this mean?](https://journals.plos.org/plospathogens/s/editorial-and-peer-review-process#loc-peer-review-history)). If published, this will include your full peer review and any attached files.


If you choose “no”, your identity will remain anonymous but your review may still be made public.


**Do you want your identity to be public for this peer review?** For information about this choice, including consent withdrawal, please see our [Privacy Policy](https://www.plos.org/privacy-policy).

Reviewer #1: No

Reviewer #2: No

Reviewer #3: No

Figure Files:

While revising your submission, please upload your figure files to the Preflight Analysis and Conversion Engine (PACE) digital diagnostic tool, [https://pacev2.apexcovantage.com](https://pacev2.apexcovantage.com/). PACE helps ensure that figures meet PLOS requirements. To use PACE, you must first register as a user. Then, login and navigate to the UPLOAD tab, where you will find detailed instructions on how to use the tool. If you encounter any issues or have any questions when using PACE, please email us at [figures@plos.org](mailto:figures@plos.org).

Response 34 – See Response 25

Data Requirements:

Please note that, as a condition of publication, PLOS' data policy requires that you make available all data used to draw the conclusions outlined in your manuscript. Data must be deposited in an appropriate repository, included within the body of the manuscript, or uploaded as supporting information. This includes all numerical values that were used to generate graphs, histograms etc.. For an example see here: <http://www.plosbiology.org/article/info%3Adoi%2F10.1371%2Fjournal.pbio.1001908#s5>.

Response 35 – CHIPseq data were submitted. See line 627. They are available to reviewers with token: uzutwkoklpennwz

Reproducibility:

To enhance the reproducibility of your results, PLOS recommends that you deposit laboratory protocols in protocols.io, where a protocol can be assigned its own identifier (DOI) such that it can be cited independently in the future. For instructions see [http://journals.plos.org/plospathogens/s/submission-guidelines#loc-materials-and-methods](http://journals.plos.org/plospathogens/s/submission-guidelines)

Response 36 – Lab protocols were not need in light of the details already provided in the Methods section.
